# SciRAPnano: a pragmatic and harmonized approach for quality evaluation of *in vitro* toxicity data to support risk assessment of nanomaterials

**DOI:** 10.3389/ftox.2023.1319985

**Published:** 2023-11-17

**Authors:** Gen Shao, Anna Beronius, Penny Nymark

**Affiliations:** Institute of Environmental Medicine, Karolinska Institutet, Stockholm, Sweden

**Keywords:** data quality, reliability, relevance, *in vitro* toxicity testing, nanomaterials, risk assessment

## Abstract

Large amounts of nanotoxicity data from alternative non-animal (*in vitro*) test methods have been generated, but there is a lack of harmonized quality evaluation approaches for these types of data. Tools for scientifically sound and structured evaluation of the reliability and relevance of *in vitro* toxicity data to effectively inform regulatory hazard assessment of nanomaterials (NMs), are needed. Here, we present the development of a pragmatic approach to facilitate such evaluation. The tool was developed based on the Science in Risk Assessment and Policy (SciRAP) tool currently applicable to quality evaluation of chemical toxicity studies. The approach taken to develop the tool, referred to as SciRAPnano, included refinement of the original SciRAP *in vitro* tool through implementation of identified NM-relevant criteria, and further refined based on a set of case studies involving evaluation of 11 studies investigating *in vitro* toxicity of nano-sized titanium dioxide. Parameters considered cover key physicochemical properties as well as assay-specific aspects that impact NM toxicity, including NM interference with test methods and NM transformation. The final SciRAPnano tool contains 38 criteria for reporting quality, 19 criteria for methodological quality, and 4 guidance items to evaluate relevance. The approach covers essential parameters for pragmatic and harmonized evaluation of NM *in vitro* toxicity studies and allows for structured use of *in vitro* data in regulatory hazard assessment of NMs, including transparency on data quality.

## 1 Introduction

Scientific toxicity data, including from alternative non-animal *in vitro* methods should be systematically reused to the extent possible during risk assessment processes (More et al., 2021b; EFSA, 2021). This is particularly critical and urgently needed in the nanosafety community, where large amounts of complex and multidisciplinary safety data have been generated, but harmonized strategies for data reuse are currently limited (Jeliazkova et al., 2021; Ji et al., 2021). Nanosafety addresses the risks of nanomaterials (NMs), which differ markedly from traditional chemicals, since variations of physicochemical properties of NMs challenges the identification of specific features driving hazard (Fadeel et al., 2018). Even though the general health risk assessment paradigm and principles used for conventional chemicals are also applicable for NMs (OECD, 2022a), NMs risk assessment should be adapted to address the complexity associated with their identity, and their biological and environmental behaviors (Laux et al., 2018). Hence, several frameworks and tools specifically for risk assessment of NMs (in some contexts referred to as nanoforms) have been proposed (Hristozov et al., 2012; Stone et al., 2014; Bos et al., 2015; Hristozov et al., 2016b; Dekkers et al., 2016), with the aim of structured data collection and evaluation, considering their unique physicochemical properties and distinct behavior (Hristozov et al., 2016a).

However, among the available risk assessment approaches, the highly variable levels and techniques used for physicochemical and biological characterization of NMs across different studies, is considered the main bottleneck for the assessment (Laux et al., 2018). Thus, there is a need for methods that facilitate systematic evaluation of data quality and relevance to inform efficient risk assessment for regulatory purposes (e.g., decision-making relating to restriction/approval of chemicals) (Nymark et al., 2017). In the regulatory context, quality of data is commonly referred to as the *reliability* of data (Molander et al., 2015). Reliability entails the study’s inherent scientific quality, demonstrated by the robustness of the methods used, reproducibility of the results and adequate description of the study (OECD, 2002). Data *relevance* describes whether a set of data are relevant for evaluating the health risks that are being assessed, i.e., relates to a specific problem formulation (Hardy et al., 2017). In general, the evaluation of “reliability” and “relevance” of toxicity studies is an integral part of the weight-of-evidence (WoE) process in regulatory chemical risk assessment context (Hardy et al., 2017).

Nevertheless, challenges regarding the evaluation of data reliability and relevance remains in the nanosafety community (Savage et al., 2019). Firstly, harmonized data quality assessment approaches are lacking, impeding both adequate health risk assessment of NMs and the reuse of data from alternative methods for risk assessment. The lack of valid experimental protocols and models for regulatory decisions, and difficulties in identifying key physicochemical properties of NMs to predict human hazards as well as reliable Test Guidelines for use under the Mutual Acceptance of Data has hindered the use of nano-derived toxicity data (OECD, 2016). To promote the efficient use of nanotoxicity data, evaluation of data quality should be performed in a transparent and structured way. Secondly, differences in use of terminology within nanosafety research and chemical risk assessment approaches have caused confusion. In addition to data quality (reliability) and relevance, the term “completeness” is also a specific (meta)data requirement to serve the intended purpose in the nanosafety community due to the necessity of extensive physicochemical characterization of NMs before and during toxicity testing (Robinson et al., 2016). (Meta)data should be “complete”, illustrated by the adequate characterization of the test NM in both physicochemical and biological aspect. However, the definition of “completeness” overlaps with data “quality” (Robinson et al., 2016). Thus, there is a need to clarify the terminology to support risk assessment of NMs.

Science in Risk Assessment and Policy (SciRAP) is a freely available online platform (www.scirap.org) which contains different tools to evaluate the reliability and relevance of *in vivo*, *in vitro* and ecotoxicity data for hazard and risk assessment of chemicals. The tools are structured around sets of predefined criteria for evaluating reliability, based on requirements and recommendations in OECD Test Guidelines, OECD Guidance Documents (e.g., Guidance Document for Describing Non-Guideline *In Vitro* Test Methods–GIVIMP (OECD, 2017)), as well as previous methods such as the Klimisch method (Klimisch et al., 1997) and the ToxRtool (Schneider et al., 2009). The SciRAP tools have been rigorously tested through external review by experts and subsequently refined, providing high scientific soundness and applicability in the regulatory setting (Molander et al., 2015; Roth et al., 2021). Reliability evaluation using the SciRAP approach consists of evaluating reporting quality (RQ) and methodological quality (MQ) separately, and is also explicitly separated from evaluation of relevance. The aim is to facilitate structured and transparent use of toxicological data in regulatory risk assessment of chemicals (Molander et al., 2015). Apart from the evaluation of reliability and relevance, SciRAP provides reporting checklists for researchers with a set of items that is necessarily included in the test report for ensuring sufficient detail and transparency (Molander et al., 2015).

The MQ and RQ of an evaluated study or dataset are presented quantitatively, with a numerical score, and qualitatively with a set of color-coded Excel charts. The output of the evaluation of relevance is presented as a qualitative color profile. The SciRAP method for evaluating *in vivo* toxicity studies was first published in 2014 and further refined in 2018 (Molander et al., 2015; Beronius et al., 2018). Recently, the SciRAP approach was also refined to enable evaluation of data quality of *in vitro* toxicity and was published and available online in 2021 (Roth et al., 2021). The tool also includes criteria for evaluation of ecotoxicity studies on NMs (Hartmann et al., 2017). However, the criteria for *in vitro* studies are not optimized for NM-specific context due to the unique requirements on physicochemical characterization (Roth et al., 2021).

Different from previous data quality assessment approaches such as the Klimisch method (Klimisch et al., 1997) which gives higher scores to studies performed with internationally standardized methods by default, SciRAP facilitates structured evaluation for non-standard studies. This is especially useful for most NMs’ toxicity studies, since availability of standardized test guidelines suitable for NM testing are currently limited (Rasmussen et al., 2016), despite the progress made in validating methods and updating test guidelines for NMs risk assessment (OECD 2018b; OECD 2018a; Gao and Lowry, 2018; OECD, 2022b). Apart from the scarcity of “standard methods” within the nanosafety community, it has been pointed out that OECD Test Guidelines revised for toxicity testing of NMs still result in new challenges for both scientists and for quality assessment when conducting GLP (good laboratory practice) studies (Lee et al., 2021). Therefore, there is an urgent need for inclusion of non-standard studies with high quality and relevance for NMs risk assessment, indicating that SciRAP is urgently needed for the NM-specific context.

The aim of the present study was to refine the SciRAP criteria towards applicability for evaluating reliability (reporting and methodological quality) and relevance of *in vitro* toxicity data for NM in support of both research and risk assessment. A number of previously published efforts and reviews aimed at identifying key physicochemical parameters relevant to quality assessment of nanosafety data were utilized as a starting point for refinement of the SciRAP criteria, including documents from EU-funded projects (caLIBRAte, 2023; GRACIOUS, 2023; GUIDEnano, 2023), as well as reviews and analyses of existing standards, regulatory guidelines and other NM-specific recommendations (ECHA, 2008; ISO, 2012; Rasmussen et al., 2016; Elberskirch et al., 2022). Finally, another aim of the study was to clarify the terminology utilized within nanosafety when referring to data quality and relevance (e.g., “completeness”) in relation to the terminology used within the regulatory context.

## 2 Methods and materials

### 2.1 Terminology

In order to clarify the diverse terminologies, evaluation of the interrelation among “reliability”, “quality”, “reporting quality”, “methodological quality”, and “completeness” was performed utilizing the terminology applied in WoE assessment approaches as a reference (Hardy et al., 2017). Details of the literature that were used to define the above terms are shown in Supplementary Table S1A1.

### 2.2 Literature search for the development of SciRAPnano

To develop the NM-applicable reliability assessment criteria and relevance items, several existing approaches and other guidance documents were reviewed. These materials were used to identify the critical parameters and topics that needed to be included in the NM-specific reliability and relevance evaluation approach. Details are shown in Supplementary Table S1A2 **.**

#### 2.2.1 Key physicochemical parameters

Diverse reports and publications from the EU-funded projects caLIBRAte (caLIBRAte), GUIDEnano (GUIDEnano), and GRACIOUS (GRACIOUS), as well as from the OECD, were used as a basis to identify key parameters to include in the current study.

The caLIBRAte quality assessment method involves the evaluation of relevance, reliability, and completeness of nanotoxicity data in the regulatory context (Nymark et al., 2017). The GUIDEnano hazard assessment strategy, developed for industrial stakeholders, provides a scoring system for evaluating the test design and reporting of NMs toxicity studies, including the physicochemical properties that need to be characterized and reported (Fernández-Cruz et al., 2018). GRACIOUS has identified the key physicochemical properties that are critical to be included in NMs’ toxicity study reporting, involving the physicochemical properties required by REACH for nanoform identification and physicochemical properties recommended by ECHA for grouping and read-across (Robinson et al., 2016). In addition, OECD WPMN has published the *Physical-chemical Decision Framework to Inform Decisions for Risk Assessment of Manufactured Nanomaterials*, to clarify requirements and reduce uncertainty in the applicability of testing and measurements for resolving knowledge gaps (OECD, 2019). The physicochemical properties involved in this framework were also considered to be integrated into the approach.

#### 2.2.2 Consideration of additional physicochemical parameters

Elberskirch et al. have analyzed existing standards, regulatory guidelines (e.g., OECD Test Guidelines), and other NM-specific guidelines to create a minimum information table (MIT) which is considered necessary to be reported in nanotoxicity testing, including a total of 300 parameters (Elberskirch et al., 2022). Besides the key physicochemical parameters covered by the other central documents described above, further NM-specific physicochemical properties within the second module (material information) of the MIT were considered for inclusion in SciRAPnano.

#### 2.2.3 NM-specific aspects of *in vitro* toxicity studies

Apart from the physicochemical properties, other NM-specific recommendations and considerations in designing and performing *in vitro* nanotoxicity studies were reviewed. In this step, guidance documents from international organizations (e.g., OECD, ISO), regulatory bodies (e.g., ECHA, EFSA), and non-regulatory academic literature were included.

### 2.3 Development of SciRAPnano *in vitro* tool v1.0

The original SciRAP criteria and items for assessment of reliability (both RQ and MQ) and relevance of *in vitro* studies provided the basis for developing the NM-applicable evaluation approach. Hence, the key physicochemical properties of NM and other NM-specific aspects identified in the previous step were integrated into the original SciRAP *in vitro* criteria. The existing SciRAP criteria were reviewed and adjusted in terms of both content and wording to be applicable to NM-data. The newly generated RQ and MQ criteria and relevance items of SciRAPnano *in vitro* tool version 1.0 (v1.0) are included in Supplementary Table S1.

### 2.4 Case study for testing SciRAPnano v1.0 and refinement to v2.0

The SciRAPnano *in vitro* tool v1.0 was tested and evaluated through assessment of a set of selected *in vitro* studies focusing on titanium dioxide (TiO_2_) nanomaterials.

#### 2.4.1 Selection of TiO_2_ studies

In order to allow for a controlled and harmonized evaluation of the tool, studies were selected based on the following considerations.

a) The study should evaluate *in vitro* toxicity induced by nano-sized TiO_2_
b) Together the selected studies should cover different types of toxicities and test systems in order to test the applicability to various study results
c) Studies with complex study design and mixtures were avoided.
d) Only studies published within the past 10 years were included
e) The selected studies should cover various levels of reporting regarding physicochemical properties, based on preliminary judgement

Based on the above considerations, 11 studies were selected. Details are shown in Supplementary Table S2B1.

#### 2.4.2 Evaluation of the selected studies

The reliability and relevance of the studies were evaluated according to the proposed v1.0 RQ and MQ criteria, and relevance items. The evaluation was done manually using Excel to consolidate the results and to further analyze the soundness of each criterion (or item) across studies. For each study, the quality criteria were judged as “fulfilled, F” “partially fulfilled, PF” “not fulfilled, NF” “not determined, ND”, whereas the relevance items were judged as “directly relevant, DR” “indirectly relevant, IR” “not relevant, NR” “not determined, ND”. Evaluation of all studies in the case study was carried out by one evaluator.

#### 2.4.3 Refinement of SciRAPnano v1.0 into v2.0

The evaluation of the case studies supported further refinement and development of SciRAPnano v1.0 into v2.0 based on the following goals: i) to obtain understanding of the general quality of recent nanotoxicity studies, especially the level of physicochemical characterization of the selected NM, ii) to identify potential refinement needs in the practical implementation of each newly proposed SciRAP criteria and item, iii) to identify potential overlaps or interconnection among criteria, and iv) to evaluate the overall adequacy and consistency of the whole approach.

## 3 Results

### 3.1 Clarifying terminology

The interrelationship between the terms “reliability”, “relevance” and “completeness” was clarified utilizing the structure and terminology of the WoE process as an example of common terminology used within regulatory risk assessment processes (Hardy et al., 2017; SHEER, 2018). The WoE process is initiated by a problem formulation process, which is coupled to the setting of a clear definition of the minimum data required to support health risk assessment. Problem formulation is followed by three steps of evidence assembling, weighing and integration to conclude the overall assessment (Hardy et al., 2017). During weighing of evidence, reliability and relevance of each single study (single evidence) is evaluated in order to be used as a basis for the assessment. It should be noted that the problem formulation sets the basis for the evaluation of data relevance. The definitions of “reliability” and “relevance” within the WoE process are shown in Table 1. During integration of the evidence, (meta)data should be curated and integrated in line with the original minimum data required during the setting of the problem, which can be considered criteria for completeness, in order to be sufficient for the specific risk assessment scenario at hand. The definition of “completeness” as guided by the WoE process is shown in Table 1.

**TABLE 1:** List of terminology used in SciRAP and how “completeness” relates to the SciRAP terminology.

Terminology	Definition
Reliability	In this project, “reliability” is synonymous to the term “quality”, as it is used in the context of toxicity data for NMs. It covers the study’s inherent scientific quality, demonstrated with the robustness of the methods used, reproducibility of the results and adequate description of the study. Thus, the evaluation of reliability is independent from the context of data application. Reliability consists of reporting quality (RQ) and methodological quality (MQ) of NMs’ physicochemical characterization and biological toxicity assessment
Reporting quality (RQ)	Describes the extent to which the description (reporting) of the design, methodology, conduct, and analysis of a study is adequate and transparent
Methodological quality (MQ)	Describes the extent to which the design and conduct of a study is sound and appropriate to generate reliable and reproducible results
Relevance	Describes the extent to which the data are relevant for answering a specific assessment question and for the assessment at hand. Relevance evaluation is thus dependent on the specific hazard or risk assessment context
Completeness	(Meta)data completeness means the tested NM should be fully characterized, in both physicochemical and biological aspect, under a specified set of experimental conditions and time points. Thus, the experimental details and associated results are supposed to be adequately described, as well as the raw data, processed data, or derived data from the assays used for NM characterization should be available. It should be noted that “completeness” is highly dependent upon the questions posed of the data or the intended use (i.e., with the specific endpoint focus). In other words, “completeness”, in the third step of WoE process, answers the question “in the generated dataset, do we have enough relevant (meta)data to perform the risk assessment?“. For instance, do we have enough physicochemical data? Do we have enough hazard data relating to the interested endpoint? Do we have bio-nano interaction data, etc. In the second step of WoE process, RQ (i.e., metadata for each single study), MQ as well as relevance of the single study for the intended use (i.e., risk assessment with focus on a specific endpoint) was assessed. Therefore, (meta)data completeness can partly refer to the “reporting quality” of the study and “relevance” in step 1. (“Methodological quality” is not included in the meaning of completeness since the latter emphasizes the availability of (meta)data instead of their intrinsic correctness (i.e., method validation, appropriateness of test system, etc.).)

### 3.2 SciRAPnano *in vitro* tool v1.0

SciRAPnano v1.0 provided the draft of criteria (items) development, based on the newly identified physicochemical properties and other NM-specific aspects in experimental design, performance and reporting, as described in the following.

#### 3.2.1 Eleven key physicochemical parameters

Eleven key physicochemical parameters were identified based on the literature review, including size, size distribution, crystallinity, shape, surface chemistry, surface area/specific surface area, surface charge, agglomeration/aggregation, stability, solubility and dissolution rate (shown in Table 2). In addition, Table 3 shows how key physicochemical properties dictate the NM’s interaction with biological system and how these parameters interlink with each other. These parameters were included among the RQ criteria within SciRAPnano. The terminology used for formulating the RQs corresponded to the eNanoMapper ontology (Hastings et al., 2015). Composition and purity were already covered by existing SciRAP criteria and were not added to the RQ. It should be noted that dustiness, density, surface hydrophobicity, free radical generation capacity, conduction band energy level, corrosivity were only mentioned once in existing minimum information checklists and thus regarded as low priority at this point.

**TABLE 2:** Overview of physicochemical properties list in different literature and the result of the identified key physicochemical properties ^a^.

caLIBRAte D5.3 (Nymark et al., 2017)	GUIDEnano^b^ (pristine NM)	GUIDEnano^b^ (NM in the exposure medium)	GRACIOUS^c^	OECD physicochemical decision framework^d^	Identified key physicochemical properties
Size/size range	Size	Size	Particle size ^prio^		**Size**
Size distribution				Particle Size Distribution	**Size distribution**
Atomic structure/crystallinity			Crystallinity ^prio^	Crystallinity	**Crystallinity**
Composition			Composition ^prio^	Chemical Composition and Impurities	***Composition***
Shape/morphology	Shape		Partical shape ^prio^	Shape	**Shape**
Surface chemistry			Surface chemistry ^prio^	Surface chemistry, coating, functionalisation	**Surface chemistry**
Surface area	Surface area		Specific surface area ^prio^	Specific Surface Area	**Surface area/Specific surface area**
Surface charge	Surface charge	Surface charge	Surface charge	Charge/zeta potential	**Surface charge**
Purity					***Purity***
Agglomeration/aggregation state				Agglomeration/Aggregation in relevant media	**Agglomeration/Aggregation**
Stability (solubility, dissolution rate)			Water Solubility	Dissolution rate in relevant media	**Stability**
					**Solubility/dissolution rate**
	Other information^e^	Other information^f^	Dustiness	Free radical generation capacity	***Low priority parameters***
			Density		
			Surface hydrophobicity	Conduction band energy level	
				Corrosivity	

^a^ the aim is to identify key physicochemical properties of tested NMs, providing potential parameters for RQ, within SciRAPnano.

^b^ in GUIDEnano, the required nanomaterial characterization parameters are separated in two subsets: A: pristine nanoparticle; B: Nanoparticle in the exposure medium. Parameters included in this sheet are from both subsets (Fernández-Cruz et al., 2018).

^c^ in GRACIOUS, composition, crystallinity, particle size, particle shape, surface chemistry and specific surface area are considered to be “priority properties” ^prio^; particularly in the context of REACH (Comandella et al., 2020).

^d^OECD, Physical-chemical Decision Framework to Inform Decisions for Risk Assessment of Manufactured Nanomaterials (OECD, 2019).

^e^ “other information” involves crystal structure, solubility, magnetic properties, acidity/basicity, redox potential, catalysis photosensitivity, hydrophobicity, radical production capacity, etc.

^f^ “other information” involves ion release, solubility, shape, etc.

**TABLE 3:** Key physicochemical properties of NMs and their link to toxicity.

NMs’ key physicochemical parameters	Influence on toxicity
Size and size distribution	Size of NMs plays a vital role in their toxicity. Owing to the small size, NMs are capable to interact with and translocate across biological barriers (e.g., placental barrier, air-blood barrier, blood brain barrier) Wang et al. (2020). Decreasing NMs’ size indicates increasing specific surface area and the percentage of surface atoms. Hence, higher probability that NMs would encounter biological molecules as well as the formation of valence band holes and conduction band electrons would result in stronger reactivity of NMs Yin et al. (2015). Further, the size of NMs may determine the site of their accumulation in organs. For example, ingested NMs smaller than 20 nm have been shown to accumulate in the kidney whereas NMs with the 20–100 nm size range tend to deposit in the liver Wang et al. (2020). It should be noted that the size of NMs is not an intrinsic property but varies (i.e., different size distribution) through dissolution, precipitation, and agglomeration processes
Crystallinity	The crystallinity of NMs indicates their degree of structural order. In a NM crystal, the arrangement of atoms or molecules is consistent and repetitive. Crystallinity is of great importance in NMs’ behavior in cellular environment. For instance, compared to the rutile form, amorphous form of TiO2 generates higher level of intracellular reactive oxygen species (ROS) Jiang et al. (2008)
Shape	The shape of NMs involves their geometry and dimensions, which impacts the cellular uptake, translocation and toxicity of NMs. For example, Rigid, long, high-aspect ratio (needle-like) NMs would undergo incomplete macrophage uptake (also known as frustrated phagocytosis) when NMs are longer than the macrophage diameter Palomäki et al. (2011), indicating the impaired clearance from the lung. In addition, 2-dimension NMs with high-aspect ratio (i.e., greater lengths relative to diameters) would exhibit geometry-dependent cellular interactions, demonstrated with spontaneous cell membrane penetration initiated at near-atomically thin edges Khan et al. (2019)
Dissolution rate/solubility and surface chemistry	Dissolution rate is the rate that ions or molecules are released from the surface of NMs into the surrounding liquid medium (2). Solubility refers to the proportion of a solute in a solvent with a saturated state More et al. (2021a)
	The surface chemistry involves hydrophobicity, residual acid or base content, coating, functionalization, defect, etc., and is strongly related to the dissolution of NMs as well as the mode and type of interactions with biotic systems. Surface hydrophilicity of charged NMs may increase their ability to be suspended in liquid medium. High surface curvature, and exposed surface atoms or molecules would increase dissolution and release of ions from metallic or metal oxide NMs. The ions released from the surface of metal NMs can be toxic. For instance, Ag ions dissolved from Ag nanoparticles are shown to inhibit carbonic anhydrase and Na+/K + ATPase activity Scown et al. (2010). Further, it has been investigated that the surface facet (i.e., arrangement of metal atoms) may influence the activation of molecular oxygen on metal surface, determining the generation of ROS Pal et al. (2012). Surface defects may also expose electron donor/acceptor active groups that donate an electron to molecular oxygen, generating superoxide anions and producing the highly reactive hydroxyl radical, and thus leading to toxicity Nel et al. (2006)
(Specific) surface area	Specific surface area (SSA) is defined as the total surface area of a material per unit of mass (with units of m2/kg or m2/g) or volume (units of m2/m3 or m−1). Since nano-scale materials have a higher surface-to-mass (volume) ratio, larger amount of exposed surface molecules or atoms would expose surface defects, and dangling chemical bonds that enhance chemical and redox reactivity. Hence, higher SSA is responsible for increased surface reactivity, increased adsorption of chemicals, strong catalytic activity and enhanced dissolution rates
Surface charge, agglomeration/aggregation and stability	In a colloidal suspension, surface charge is usually quantified by zeta potential, illustrated by the electric potential generated between the NMs’ surface and the dispersion medium Selvamani, (2019). Surface charge has an evident impact to the stability of NMs in suspension by particle–particle attachment and thus altering their agglomeration (i.e., weak bound of particles) and aggregation (i.e., strong bound or fusion of particles) behavior. Hence, NMs sedimentation caused by agglomeration or aggregation may influence the dose metrics as the delivered dose or cellular uptake would be significantly different from the nominal dose, indicating the potential misinterpretation of dose-response relationship. Apart from the surface charge, stability of NMs is also determined by their size, density, electronic structure, and morphology. In addition, NMs may adsorb molecules from the surrounding environment that increase the suspension stability (e.g., serum albumin) Nel et al. (2009)
	Surface charge of NMs also plays an important role in interactions with cellular structure. Cationic particles cause more pronounced disruption of plasma-membrane integrity than anionic counterparts Fröhlich, (2012). Positively charged NMs tend to interact with negatively charged molecules (e.g., DNA)
	It should be noted that stability was not included among the RQ since this parameter was considered to overlap with agglomeration state and solubility

#### 3.2.2 Four additional physicochemical properties

The following physicochemical properties were considered as lower priority than key physicochemical properties, since they were only mentioned once among all selected resources (see Table 2.) but were covered by the open criterion “other information” in SciRAPnano v1.0 RQ criteria list (RQ #34).

1. Delivery form: Powder or suspension.
2. Dustiness: This parameter is regarded as low priority in the first step. However, it is relevant for NMs’ exposure via air (particularly by inhalation) and required by REACH (Annex VII) (EC, 2018).
3. Viscosity: Viscosity of the media after sample preparation.
4. Labelling: Nanomaterials are frequently labelled to track their biological interactions (e.g., fluorescent probes, radiolabeling). The labeling details should be reported, if applicable.

#### 3.2.3 Eight critical aspects of *in vitro* toxicity testing of nanomaterials

Based on the literature review, the following 8 aspects were considered NM-specific and may affect the study result in NMs’ toxicity testing regarding the study design and performance and are mostly incorporated into RQ and MQ criteria.

1. Sample preparation method and the dispersion stability
2. Transformation and temporal changes of the NM
3. Dispersant and/or stabilizers
4. Dose metrics
5. The reduced or excessive delivery of NMs into test systems due to the agglomeration status of NMs
6. NMs interference with the test method
7. NM physicochemical properties characterization
8. Added substances to the test system and potential contamination to the test system

#### 3.2.4 SciRAPnano v1.0 reliability criteria

The above parameters and aspects were formulated and integrated in the original SciRAP *in vitro* section, generating the first version of SciRAPnano *in vitro* quality assessment approach. The details about the new criteria and rewording of existing SciRAP RQ and MQ criteria are shown in Supplementary Tables S1A3, A4. RQ#5-15, MQ#2, MQ#3 MQ#10 and MQ#16 are new criteria and not present in the original SciRAP *in vitro* section. MQ#4, MQ#5, MQ#11, MQ#12, MQ#13 were reworded owing to the identified NM-specific aspects, based on the original SciRAP criteria. In addition, the original SciRAP MQ#2 criterion: *It was likely that the test compound was soluble at the concentrations used* was deleted since this is not relevant for NMs, which are not in general soluble in common solvents.

#### 3.2.5 SciRAPnano v1.0 relevance item(s)

The four relevance items in the original SciRAP *in vitro* tool are i) the identity of the tested substance; ii) the test system used; iii) the endpoint studied; and iv) the concentrations used. These items were not changed for NM-specific reasons. However, since NMs may transform, the test item in toxicity studies may not be relevant to the risk assessment of the nanoform of interest. Hence, this aspect was added to the guidance of the first relevance item (the identity of the test item). Details are provided below in chapter 3.5.

### 3.3 Case studies

An overview of ratings for each of the 11 studies evaluated in the test round for RQ and MQ reliability criteria, and relevance items, is shown in Tables 4-6, respectively.

**TABLE 4:** Results of RQ evaluation of 11 selected studies by SciRAPnano criteria. Each column represents the evaluation of an individual study; rows represent individual criteria. Green cells indicate criteria judged as “fulfilled” (F), yellow cells indicate criteria judged as “partially fulfilled” (PF), red cells indicate criteria judged as “not fulfilled” (NF), and grey cells indicate criteria left as “not determined” (ND). Details of the case studies are available in Supplementary Table S2B1.

Reporting quality criteria		Study 1	Study 2	Study 3	Study 4	Study 5	Study 6	Study 7	Study 8	Study 9	Study 10	Study 11
Test item and controls	1	F	F	F	F	F	F	F	F	F	F	F
	2	F	NF	NF	NF	F	NF	NF	F	F	F	F
	3	F	NF	F	NF	F	F	F	F	F	F	NF
	4	F	F	F	F	F	F	F	F	F	F	F
Physicochemical properties of the test item	5	F	F	F	F	F	F	F	F	F	F	F
	6	NF	F	NF	F	F	F	F	NF	NF	F	NF
	7	F	F	F	NF	F	NF	NF	F	F	F	F
	8	F	NF	F	NF	F	F	NF	F	F	F	NF
	9	F	NF	NF	NF	F	NF	NF	F	NF	NF	NF
	10	F	F	F	NF	F	F	NF	F	NF	NF	F
	11	NF	F	NF	NF	F	NF	NF	F	F	NF	F
	12	NF	NF	NF	NF	NF	NF	NF	NF	NF	NF	PF
	13	NF	NF	NF	NF	NF	NF	NF	NF	NF	NF	NF
	14	F	PF	F	NF	F	F	F	F	NF	NF	NF
	15	F	NF	F	F	F	F	F	F	F	F	PF
Test System	16	F	F	F	F	F	F	F	F	F	F	F
	17	F	F	F	F	F	F	F	F	F	F	F
	18	NF	NF	NF	NF	NF	NF	NF	NF	NF	NF	NF
	19	PF	F	PF	NF	NF	NF	NF	NF	NF	NF	ND
	20	F	F	F	F	F	F	NF	F	F	F	F
	21	F	F	F	F	F	F	F	F	NF	F	F
	22	F	NF	NF	NF	NF	NF	NF	NF	NF	NF	NF
Administration of test compound	23	F	F	F	F	F	F	F	F	F	F	F
	24	F	F	NF	NF	F	F	F	F	F	F	ND
	25	F	F	F	F	F	F	F	F	F	F	F
	26	F	F	F	NF	F	NF	F	F	F	F	F
Data collection and analysis	27	F	NF	PF	NF	F	PF	F	F	F	F	F
	28	F	PF	NF	NF	F	F	PF	F	NF	NF	F
	29	F	F	F	F	F	F	PF	F	F	F	F
	30	F	F	F	NF	F	F	F	F	F	F	F
	31	F	F	F	F	F	F	F	F	F	F	F
Funding and competing interests	32	F	F	F	F	F	F	NF	F	F	F	F
	33	F	F	F	F	F	F	F	F	F	F	F

**TABLE 5:** Results of MQ evaluation of 11 selected studies by SciRAPnano criteria. Each column represents the evaluation of an individual study; rows represent individual criteria. Green cells indicate criteria judged as “fulfilled” (F), yellow cells indicate criteria judged as “partially fulfilled” (PF), and red cells indicate criteria judged as “not fulfilled” (NF). Details of the case studies are available in Supplementary Table S2B1.

Methodological quality criteria		Study 1	Study 2	Study 3	Study 4	Study 5	Study 6	Study 7	Study 8	Study 9	Study 10	Study 11
Test item and controls	1	F	PF	PF	NF	F	PF	PF	F	F	F	F
	2	PF	PF	F	NF	F	F	F	F	PF	F	PF
	3	F	NF	F	NF	F	NF	F	NF	PF	PF	PF
	4	F	F	F	F	F	F	F	F	F	F	F
	5	F	F	PF	PF	F	F	NF	F	F	F	PF
	6	PF	NF	F	NF	F	F	NF	F	NF	NF	NF
Test System	7	F	F	F	F	F	F	F	F	F	F	F
	8	F	F	F	PF	F	F	F	F	NF	F	F
Administration of test compound	9	F	F	F	F	F	F	F	F	F	F	F
	10	F	PF	PF	NF	F	F	F	NF	F	F	PF
	11	F	F	PF	PF	F	PF	NF	F	PF	F	F
	12	F	F	F	NF	PF	PF	F	F	PF	PF	PF
Data collection and analysis	13	F	F	PF	F	F	F	F	F	F	F	PF
	14	F	F	F	F	F	PF	F	F	F	F	F
	15	F	NF	PF	PF	F	F	F	F	F	F	F
	16	PF	NF	F	NF	PF	PF	PF	PF	PF	NF	NF
	17	F	F	F	F	F	PF	PF	F	F	F	F
	18	F	F	F	PF	F	PF	NF	PF	NF	F	F
	19	F	F	F	PF	F	F	F	F	F	F	F

**TABLE 6:** Results of relevance evaluation of 11 selected studies by SciRAPnano items. Each column represents the evaluation of an individual study; rows represent individual criteria. Green cells indicate items judged as “directly relevant” (DR), whereas yellow cells indicate items judged as “indirectly relevant” (IR). No items were judged as “not relevant” in this case. Details of the case studies are available in Supplementary Table S2B1.

Relevance items	Study 1	Study 2	Study 3	Study 4	Study 5	Study 6	Study 7	Study 8	Study 9	Study 10	Study 11
The identity of the tested item	DR	DR	DR	DR	DR	DR	DR	DR	DR	DR	DR
The test system used	DR	DR	IR	IR	IR	DR	DR	DR	DR	DR	DR
The endpoint studied	DR	DR	DR	DR	DR	DR	DR	DR	DR	DR	DR
The concentration used	DR	IR	DR	DR	DR	IR	IR	DR	DR	DR	DR

For RQ, the least reported parameters among all studies were “#12 water solubility of the test item”, “#13 dissolution rate of the test item” “#18 metabolic competence of the test system”, and “#22 measures taken for avoiding or screening for contamination” (Table 4). In addition, the whole “physicochemical properties of the test item” section was poorly reported, with #5 “size” being the only fully reported parameter within all 11 physicochemical properties across all studies.

For MQ, “#16 NMs physicochemical properties characterization was adequately performed at least ‘as received’, ‘as administered’ and ‘after administration or *in situ*’” is the least fulfilled criterion, indicating the poor characterization of physicochemical properties of the tested NM (Table 5). This finding corresponds to the inadequately reported physicochemical properties (cf. Table 4). In addition, the NM interference of the test item with the test condition (MQ#12) was not fully considered in most of the studies. Besides, due to the agglomeration of nano-TiO_2_ dispersion, MQ#3 which assesses whether a stable dispersion was maintained, was scarcely fulfilled.

For the purpose of relevance evaluation, a generic problem was formulated as follows: “human health risk assessment of nano-size TiO_2_ in real-life exposure level”. Items #1 and #3 were both judged as “directly relevant” for all studies which investigates the toxicity of nano-TiO_2_ for different endpoints (Table 6). In contrast, many of the studies do not explain the rationale for the chosen exposure level, making it difficult to evaluate item #4 (the concentration used).

### 3.4 SciRAPnano *in vitro* tool v2.0

#### 3.4.1 SciRAPnano reliability criteria (v2.0)

Based on the refinement needs identified in the case study, SciRAPnano *in vitro* tool v1.0 was refined to SciRAPnano *in vitro* tool v2.0, which involves 38 criteria for RQ and 19 criteria for MQ. The full lists of all criteria are shown in Table 7 and Table 8. In addition, a revised guidance for the MQ section is presented in Supplementary Table S3C1.

**TABLE 7:** The SciRAPnano *in vitro* tool 2.0 RQ criteria. The left column is the full list of RQ criteria. The right column lists NM-specific parameters/aspects identified in the literature research (see Methods 2.2) and the refinement of SciRAPnano v1.0 (see Method 2.4). “/” indicates that the corresponding criteria remains the same as in the original SciRAP *in vitro* tool.

Test item and controls	Identified NM-associated parameters/aspects
1. The chemical name or other identification, such as CAS-number, of the test item was given	—
2. The purity of the test item was stated or is traceable according to information given regarding manufacturer and lot/batch number. In case of mixtures, the composition of different constituents was stated	—
3. The solvent (vehicle) was described	—
4. It was stated that a solvent (vehicle) control was included	—
**Physicochemical properties of the test item**	
5. The size of the test item was stated	Size
6. The size distribution of the test item was stated	Size distribution
7. The crystallinity of the test item was stated	Crystallinity
8. The shape of the test item was stated	Shape/morphology
9. The surface chemistry of the test item was stated	Surface chemistry
10. The surface charge of the test item was stated	Surface charge
11. The surface area of the test item was stated	Surface area/specific surface area
12. The water solubility or dissolution rate of the test item was described	Stability/water solubility or dissolution rate
13. The agglomeration/aggregation of the test item was stated	Agglomeration/aggregation
14. The sample preparation methodology of the test item was stated	Sample preparation method
15. The dispersion stability of the test item was stated	Dispersion stability
16. The transformation of the test item or temporal changes of its physicochemical properties has been described	Transformation and temporal changes of NM
**Test System**	
17. The test system (e.g., cell line/cells/tissue/organ/embryo/sub-cellular fractions) was described	—
18. The source of the test system was stated	—
19. The metabolic competence, i.e., competence of the test system to metabolize the test compound into an active metabolite was described	—
20. The number of cell passages of the cell line used was stated. (Remove this criterion if the study was not conducted in a cell line.)	—
21. Composition of media was described, including use of serum, antibiotics, etc.	—
22. Incubation temperature, humidity, and CO2 concentration were described	—
23. Measures taken for avoiding or screening for contamination by *mycoplasma*, bacteria, fungi and virus were described	—
24. Measures taken to avoid or address the test item interference with the test method were described	Test item interference with the test method
**Administration of test item**	
25. The administered dose levels or concentrations were stated	—
26. Cell density or number of cells used during treatment was described. (Remove this criterion if the study was not conducted in a cell line.)	—
27. The duration of treatment was stated	—
28. The number of replicates per dose level/concentration or the number of times the experiment was repeated was stated	—
29. The cellular uptake or distribution of the test item was measured	The reduced or excessive delivery of NMs into test systems due to the agglomeration status of NMs
**Data collection and analysis**	
30. The tests and/or analytical methods used were sufficiently described to allow for evaluation of reliability of results	—
31. The dose metrics used were appropriately reported	dose metrics
32. The time points for data collection were stated	—
33. It was stated that the effect of the test compound on cytotoxicity was measured	—
34. All results were clearly presented	—
35. The statistical methods and software used were described	—
**Funding and competing interests**	
36. The funding sources for the study were stated	—
37. Any competing interests were disclosed or it was explicitly stated that the authors did not have any competing interests	—
**Other information**	
38. Was all information that is indispensable for evaluating the reliability of data given? This includes information on the test compound and controls, test system, study design or study performance. For example,: *delivery form; viscosity; dustiness; labelling information*	Low prioritized physicochemical properties

**TABLE 8:** The SciRAPnano *in vitro* tool 2.0 MQ criteria. The left column is the full list of MQ criteria. The right column lists NM-specific parameters/aspects identified in the literature research (see Methods 2.2) and refinement of SciRAPnano v1.0 (see Method 2.4). “/” indicates that the corresponding criteria remains the same as the original SciRAP *in vitro* tool.

Test item and controls	Identified NM-associated parameters/aspects
1. The test item or mixture was unlikely to contain any impurities that may significantly have affected the results of the study	—
2. A dispersion of the test item was created by appropriate sample preparation method	Sample preparation method
3. The transformation of the test item or temporal changes of its physicochemical properties is not expected to affect the result	Transformation and temporal changes of NM
4. An appropriate solvent (vehicle) was used that is not expected to interfere with the results of the study at the concentration used	—
5. A solvent (vehicle) control was included	—
6. The dispersant/stabilizer used is not expected to interfere with the results of the study	Dispersant/stabilizer
7. An appropriate positive control was included, and the expected result was observed from this treatment	—
**Test system**	
8. A reliable and sensitive test system (cell line/cells/tissue/organ/embryo/sub-cellular fractions) with metabolic competence, if relevant, was used for investigating the test item and endpoints	—
9. Conditions for cultivation and/or maintenance of the cell line/cells/tissue/organ/embryo (incubation temperature, humidity, CO2 concentration, media used, number of cell passages, control of contamination) were appropriate	—
**Administration of the test item**	
10. The duration of exposure was suitable for the test system and investigated endpoints	—
11. The concentrations used were suitable for the test system and investigated endpoints	—
12. The test conditions during and after exposure to the test item were suitable (media and serum used, cell density, incubation temperature, humidity, CO2 concentration)	—
**Data collection and analysis**	
13. Reliable and sensitive tests and/or analytical methods were used for investigating the endpoints	—
14. The test item is not expected to interfere with the test method so as to affect the study result	NM interference with the test method
15. Sufficient numbers of replicates or repetitions of the experiment were used to generate reliable and valid results	—
16. Measurements were collected at suitable time points in order to generate sensitive, valid and reliable data	—
17. Cytotoxicity was measured and the test item did not cause cytotoxicity that significantly affected the results	—
18. The statistical methods were clearly described and do not seem inappropriate, unusual or unfamiliar	—
**Other information**	
19. Are there any other aspects of study design, performance or reporting that influence reliability?	Added substances to the test system and potential contamination to the test system

The overview and the detailed description of the refinement and reasons behind the refinement can be found in Supplementary Table S1. Briefly, the refinement included considerations of i) harmonization between the NM-specific criteria and the original SciRAP terminology/wording as far as possible, ii) overlaps with existing criteria, iii) a need to integrate or separate NM-specific criteria from existing criteria, and iv) clarification of NM-specific terminology and their positioning in the tool (e.g., NM-specific RQ vs. MQ, interference vs. interaction).

In total, 15 RQ criteria (#5-16, #24, #29, #31) and 3 MQ criteria (#3, #6, #14) were added based on the literature review and the case study. Further, one new MQ criterion (#2) replaced the original MQ criterion (#2) in the SciRAP *in vitro* tool. It should be noted that both RQ and MQ have an open criterion for evaluators to provide other aspects apart from the existing criteria (e.g., study design, performance, and reporting) that affects the overall reliability. These functions are retained in the final SciRAPnano tool and users are recommended to use the “comment” function for each criterion and to provide the rationale behind each evaluation.

### 3.5 SciRAPnano relevance items (v 2.0)

The SciRAPnano *in vitro* tool 2.0 involves 4 items for evaluating relevance. No alteration or refinement of items formulations themselves were done in NM-specific context. However, the guidance for the first item (the identity of the test item) was revised in SciRAPnano v1.0 owing to the distinct physicochemical properties of NMs, details are shown in Table 9. Due to the generic problem formulation relating to the case studies (see section 3.3) no further refinement of the relevance items was required in v2.0 of the SciRAPnano tool.

**TABLE 9:** The SciRAPnano *in vitro* tool 2.0 relevance items. The left column is the full list of relevance items. The right column lists NM-specific parameters/aspects identified in the literature research (see Methods 2.2) and used to refine the guidance for the item. “/” indicates that the corresponding relevance item remains the same as in the original SciRAP *in vitro* tool.

Relevance items	Guidance and identified NM-associated aspects (*in italics*)
The identity of the tested substance	Consider if, based on the information given, i.e., CAS-number or similar, it can be concluded that the substance being investigated is the same as the substance subject to assessment. Consideration should also be given to the purity of the test substance and whether there may be impurities present that could significantly affect the identity of the substance. *Also, the state of the tested NM should be considered. For NMs that may transform, is the appropriate form of the substance being tested?*
The test system used	—
The endpoint studied	—
The concentrations used	—

## 4 Discussion

To facilitate structured and transparent use of *in vitro* toxicity data for regulatory hazard and risk assessment of NMs, SciRAPnano was developed to provide a pragmatic, harmonized and user-friendly approach to evaluate data reliability and relevance for regulatory purpose. The tool was developed based on the previously established SciRAP *in vitro* tool, which has rigorously been tested through internal and external review (Roth et al., 2021). The SciRAPnano *in vitro* approach is also in accordance with both the trend of Next-Generation Risk Assessment of NMs and recommendation from regulatory authorities that non-animal alternatives should be applied whenever possible to avoid animal experimentation (EC, 2009; Browne et al., 2019).

Within the EU, efforts have been made to ensure that data are of high quality in NM’s risk assessment. For example, the German-funded DaNa 2.0 project (DaNa, 2023) created a Literature Criteria Checklist which evaluates whether NMs’ physicochemical properties and experimental details are fully described in toxicological publications. In addition, Elberskirch et al. (Elberskirch et al., 2022) created a metadata schema to establish a representation standard for nanotoxicity studies, including 300 parameters in 6 modules. These measures, to a large extent, contribute to ensuring studies with high reporting quality being included for NM’s risk assessment. However, adequate data reporting may not automatically ensure data quality, and experimental design and performance also play a vital role in determining the overall reliability and relevance of studies (Grafström et al., 2021; Jeliazkova et al., 2021; Saarimäki et al., 2021). To this end, SciRAPnano allows for a pragmatic and harmonized evaluation, considering the most critical parameters in line with the characterization requirement of nanoforms under REACH and guidance documents from EU chemical regulatory bodies. Moreover, the detailed criteria in SciRAPnano contribute to ensure that central aspects for evaluating the inherent scientific quality of the study (i.e., experimental design and study performance) are considered across all studies, regardless of compliance with standardized test guidelines, which remain limited in nanosafety community. In addition, the approach is harmonized with quality assessment of chemical toxicity studies, providing a basis for comparable quality assessments between the fields, to the extent possible.

SciRAPnano enables assessors to evaluate the data in a semi-automated and structured fashion. Easy-to-follow instructions for how to fulfill MQ criteria requirements are provided. In addition, SciRAPnano users can provide comments (e.g., the rationale of each judgement) under each criterion during data evaluation providing means for transparency. Individual evaluation reports for each evaluated study can be generated and color-coding charts and quantitative scores indicate the quality of studies. In addition to risk assessors, SciRAPnano is also useful for researchers by supporting appropriate study design and sufficient reporting, and therefore improving the usefulness of data for regulatory purposes.

The presented pragmatic approach also leaves room for flexibility (i.e., expert judgement) which is particularly important in its implementation as many aspects still lack consensus within the nanosafety community. For example, owing to the complexity of the protein corona profile and the lack of understanding regarding its effect on toxicity, it remains unclear if and how the bio-corona should be taken into account in NMs’ risk assessment (Westmeier et al., 2015; Lin et al., 2017). In SciRAPnano, the “bio-corona” aspect is covered by the aspect of *transformation* (RQ#16 and MQ#3) and would be evaluated on a case-by-case basis with expert judgement. Moreover, expert judgement is also needed beyond SciRAPnano in evaluating nanosafety data quality. For instance, apart from the key parameters identified in the present study, other physicochemical properties (photocatalysis, redox potential, etc.) are also potential descriptors for NM’s toxicity (Fu et al., 2014). Some toxicity determinants should only be considered specific to the type of NM or to the adverse outcomes and thus, were not prioritized in SciRAPnano (e.g., dustiness determines the toxicity of airborne NMs, but is not related to risk from other exposure routes (Donaldson and Poland, 2013)). These potential parameters were not included in the approach but should be considered and evaluated on a case-by-case scenario.

Overall it should be noted that a pragmatic approach does not necessarily refer to complete exclusion of expert judgement, but rather aims to bring transparency, consistency and structure to the process (Wandall et al., 2007; Beronius and Ågerstrand, 2017). The SciRAPnano approach ensures that data quality and relevance assessment are systematically performed while allowing for flexibility, which is particularly important for non-standard studies, e.g., during WoE processes in risk assessment (Ingre-Khans et al., 2020).

Relating to the above, it was noted at the start of this study that the evolution of terminology within the nanosafety field has caused difficulties and unclarities associated with quality and relevance assessment of data and literature from the nanotoxicology field within regulatory risk assessment (OECD, 2022a). For example, within nanosafety, a commonly used term to indicate data requirements is “completeness”, which in general refers to the necessity of adequate physicochemical characterization of NMs in toxicity testing. The term has proved difficult to fit within the chemical regulatory context, where data should be “reliable” and “relevant”, and often causes confusion as to whether it relates to data being complete enough in order to be reliable (i.e., sufficient physicochemical characterization to reliably assess toxicity in a given test system), or complete enough in order to be relevant (i.e., sufficient physicochemical characterization to relevantly address a specific problem formulation) (Robinson et al., 2016; Hardy et al., 2017). Here, the term “completeness” of (meta)data was clarified from a regulatory perspective using the WoE assessment approach as a reference, contributing to the alignment of the nanosafety community with the regulatory context. Overall, the term was strongly associated with a dependency on the questions posed (e.g., the problem formulation in a risk assessment context) and therefore, from a regulatory perspective, related to reporting quality and relevance, but not to methodological quality (cf. Table 1). However, further effective communication is still needed among regulatory agencies, industries and nanosafety experts to progress data quality and relevance assessment for risk assessment of NMs.

## 5 Conclusion

Here we present the newly developed SciRAPnano tool for evaluation of reliability and relevance of *in vitro* studies on NMs. It is based on the previously available SciRAP tool for *in vitro* studies and covers 38 RQ criteria (involving 15 new or adjusted criteria to evaluate key physicochemical properties, sample preparation methodology, cellular uptake, dose metrics, dispersion stability, NM’s transformation and measures taken to address the potential interference) and 19 MQ criteria (involving 4 new or adjusted criteria covering NM’s transformation, dispersant, NMs’ interference with test method and sample preparation). In addition, the tool involves four relevance items, which remain unchanged from the original SciRAP *in vitro* tool, except for an addition to the guidance of the first item (the identity of the test item), which was revised based on the consideration of NMs potential for transformation. Other aspects relevant to NMs, involving delivery form, viscosity, dustiness, labelling information of NMs, as well as potential contamination within the test sample, and extra substances added to the test system, are included in the “other information” sections of the RQ and MQ criteria, respectively.

Overall, SciRAPnano allows for pragmatic, harmonized and user-friendly evaluation of data reliability and relevance in order to support structured and transparent use of toxicological data within, for example, WoE processes for risk assessment of NMs. In addition to support for regulatory risk assessment of NMs, the approach can be expected to provide extensive support to the recently proposed framework aimed to guide innovation involving chemicals and materials towards becoming Safe and Sustainable by Design (SSbD) (Caldeira et al., 2022). The framework is heavily dependent on the reuse of existing data and knowledge, indicating an urgent need for harmonized and structured data quality and relevance assessment across diverse agents, including NMs (Nymark et al., 2020).

## Data Availability

The original contributions presented in the study are included in the article/Supplementary Materials, further inquiries can be directed to the corresponding author.

## Supplementary material

SUPPLEMENTARY TABLE S1 Literature and description of SciRAPnano v1.0.

SUPPLEMENTARY TABLE S2 Overview of case studies.

SUPPLEMENTARY TABLE S3 SciRAPnano guidance for MQ criteria.
