# Supplementary material for: SciRAPnano: a pragmatic and harmonized approach for quality evaluation of in vitro toxicity data to support risk assessment of nanomaterials
Source: Front Toxicol. 2023 Nov 17;5:1319985. doi: 10.3389/ftox.2023.1319985 (PMC10691260; doi:10.3389/ftox.2023.1319985)
Supplement: Supplementary file 1 [file Table1.docx]

**Supporting information - Appendix A**

**Table A1.** List of literature used to define the terminology in this project

| **Titles of literature** | **Authors/Authority, year of publication, references** |
| --- | --- |
| Guidance on information requirements and chemical safety assessment-Chapter R.4: Evaluation of available information | European Chemical Agency (ECHA) |
| Guidance Document on the validation and international acceptance of new or updated test methods for hazard assessment | Organization for Economic Co-operation and Development (OECD 2005) |
| Guidance on the use of the weight of evidence approach in scientific assessments | European Food Safety Authority (Hardy et al. 2017) |
| Science in Risk Assessment and Policy (SciRAP): An Online Resource for Evaluating and Reporting In Vivo (Eco)Toxicity Studies | (Molander et al. 2015) |
| Testing and refining the Science in Risk Assessment and Policy (SciRAP) web-based platform for evaluating the reliability and relevance of in vivo toxicity studies | (Beronius et al. 2018) |
| Development of the SciRAP Approach for Evaluating the Reliability and Relevance of in vitro Toxicity Data | (Roth et al. 2021) |
| caLIBRAte D5.3 - Document on quality criteria for data | (Nymark et al. 2017) |
| Quality of physicochemical data on nanomaterials: an assessment of data completeness and variability | (Comandella et al. 2020) |
| How should the completeness and quality of curated nanomaterial data be evaluated? | (Robinson et al. 2016) |

**Table A2.** List of materials used for identifying physicochemical properties and other NM-specific aspects to be included in SciRAPnano *in vitro* tool.

| **Title of the materials used for identifying different types of parameters** | **Authors/Authority, year of publication, reference** |
| --- | --- |
| *Key physicochemical parameters* | |
| caLIBRAte D5.3-Document on quality criteria for data | (Nymark et al. 2017) |
| Quality evaluation of human and environmental toxicity studies performed with nanomaterials – the GUIDEnano approach | (Fernández-Cruz et al. 2018) |
| GRACIOUS-A framework for grouping and read-across of nanomaterials- supporting innovation and risk assessment | (Stone et al. 2020) |
| Physical-chemical Decision Framework to Inform Decisions for Risk Assessment of Manufactured Nanomaterials | (OECD 2019a) |
| *Other relevant physicochemical parameters* | |
| Digital research data: from analysis of existing standards to a scientific foundation for a modular metadata schema in nanosafety | (Elberskirch et al. 2022) |
| *NM-specific aspects of in vitro toxicity studies* | |
| Nanomaterials in REACH and CLP | (EFSA 2021) |
| Test No. 318: Dispersion Stability of Nanomaterials in Simulated Environmental Media | (OECD 2017) |
| Guidance document for the testing of dissolution and dispersion stability of nanomaterials and the use of the data for further environmental testing and assessment strategies | (OECD 2020) |
| Important issues on risk assessment of manufactured nanomaterials | (OECD 2022) |
| Evaluation of in vitro methods for human hazard assessment applied in the OECD Testing Programme for the Safety of Manufactured Nanomaterials | (OECD 2018) |
| Genotoxicity of manufactured nanomaterials report of the OECD expert meeting | (OECD 2014) |
| Guidance on sample preparation and dosimetry for the safety testing of manufactured nanomaterials | (OECD 2012) |
| Guiding principles for measurements and reporting for nanomaterials physical chemical parameters | (OECD 2019b) |
| Physical-chemical parameters: measurements and methods relevant for the regulation of nanomaterials | (OECD 2019c) |
| Physical-chemical properties of nanomaterials evaluation of methods applied in the OECD WPMN testing program | (OECD 2016) |
| Guidance on physico-chemical characterization of engineered nanoscale materials for toxicological assessment | (ISO 2012) |
| Recommendations for nanomaterials applicable to Chapter R7a Endpoint specific guidance | (ECHA 2017) |
| Safety assessment of titanium dioxide (E171) as a food additive | (EFSA et al. 2021) |
| The appropriateness of existing methodologies to assess the potential risks associated with engineered and adventitious products of nanotechnologies | (SCENIHR 2006) |
| In vitro approaches to assess the hazard of nanomaterials | (Drasler et al. 2017) |
| Interference of engineered nanoparticles with in vitro toxicity assays | (Kroll et al. 2012) |
| Identification and Avoidance of Potential Artifacts and Misinterpretations in Nanomaterial Ecotoxicity Measurements | (Petersen et al. 2014) |
| Influence of Ionic Strength, pH, and Cation Valence on Aggregation Kinetics of Titanium Dioxide Nanoparticles | (French et al. 2009) |
| Quality of physicochemical data on nanomaterials: an assessment of data completeness and variability | (Comandella et al. 2020) |
| Reporting Guidelines for the Preparation of Aqueous Nanoparticle Dispersions from Dry Materials - Version 2.1 | (Taurozzi et al. 2012) |

**Table A3.** The SciRAPnano v1.0 RQ criteria

| **SciRAPnano v1.0 RQ Criteria** |
| --- |
| 1. The chemical name or other identification, such as CAS-number, of the test compound was given. |
| 1. The purity of the test item was stated or is traceable according to information given regarding manufacturer and lot/batch number. In case of mixtures, the composition of different constituents was stated. |
| 1. The solvent (vehicle) was described. |
| 1. It was stated that a solvent (vehicle) control was included. |
| 1. The size of the test item was stated. |
| 1. The size distribution of the test item was stated. |
| 1. The crystallinity of the test item was stated. |
| 1. The shape of the test item was stated. |
| 1. The surface chemistry of the test item was stated. |
| 1. The surface charge of the test item was stated. |
| 1. The surface area of the test item was stated. |
| 1. The water solubility of the test item was described. |
| 1. The dissolution rate of the test item was stated. |
| 1. The agglomeration/aggregation of the test item was stated. |
| 1. The sample preparation methodology of the test item was stated. |
| 1. The test system (e.g. cell line / cells/ tissue / organ / embryo / sub-cellular fractions) was described. |
| 1. The source of the test system was stated. |
| 1. The metabolic competence, i.e. competence of the test system to metabolize the test compound into an active metabolite was described. |
| 1. The number of cell passages of the cell line used was stated. (Remove this criterion if the study was not conducted in a cell line.) |
| 1. Composition of media was described, including use of serum, antibiotics, etc. |
| 1. Incubation temperature, humidity, and CO2 concentration were described. |
| 1. Measures taken for avoiding or screening for contamination by mycoplasma, bacteria, fungi and virus were described. |
| 1. The administered dose levels or concentrations were stated. |
| 1. Cell density or number of cells used during treatment was described. (Remove this criterion if the study was not conducted in a cell line.) |
| 1. The duration of treatment was stated. |
| 1. The number of replicates per dose level/concentration or the number of times the experiment was repeated was stated. |
| 1. The tests and/or analytical methods used were sufficiently described to allow for evaluation of reliability of results. |
| 1. The time points for data collection were stated. |
| 1. It was stated that the effect of the test compound on cytotoxicity was measured. |
| 1. All results were clearly presented. |
| 1. The statistical methods and software used were described. |
| 1. The funding sources for the study were stated |
| 1. Any competing interests were disclosed or it was explicitly stated that the authors did not have any competing interests. |
| 1. Was all information that is indispensable for evaluating the reliability of data given? This includes information on the test compound and controls, test system, study design or study performance.   For example: delivery form; viscosity; dustiness; labelling information. |

**Table A4.** The SciRAPnano v1.0 MQ criteria

| **SciRAPnano v1.0 MQ Criteria** |
| --- |
| 1. The test item or mixture was unlikely to contain any impurities that may significantly have affected the results of the study. |
| 1. The transformation of the test item or temporal changes of its physiochemical properties has been considered and/or monitored. |
| 1. A stable dispersion of the test item was created and maintained by appropriate sample preparation method. |
| 1. An appropriate solvent (vehicle) was used that is not expected to interfere with the results of the study at the concentration used. Dispersant/stabilizers was used properly if needed. |
| 1. A solvent (vehicle) control was included. Dispersants/stabilizers were added if needed. |
| 1. An appropriate positive control group was included, and the expected result was observed from this treatment. |
| 1. A reliable and sensitive test system (cell line / cells / tissue / organ /embryo / sub-cellular fractions) with metabolic competence, if relevant, was used for investigating the test compound and endpoints. |
| 1. Conditions for cultivation and/or maintenance of the cell line / cells / tissue / organ /embryo (incubation temperature, humidity, CO2 concentration, media used, number of cell passages, control of contamination) were appropriate. |
| 1. The duration of exposure was suitable for the test system and investigated endpoints. |
| 1. The dose metrics used were suitable for the test item. |
| 1. The concentrations used were suitable for the test system and investigated endpoints. Transformation or agglomeration of the test item should be considered. |
| 1. The test conditions during and after exposure to the test compound were suitable (media and serum used, cell density, incubation temperature, humidity, CO2 concentration, NMs incompatibilities and interference). |
| 1. Reliable and sensitive tests and/or analytical methods were used for investigating the endpoints, the physiochemical properties of the tested NM or the potential interference has been taken into account. |
| 1. Sufficient numbers of replicates or repetitions of the experiment were used to generate reliable and valid results. |
| 1. Toxicological endpoints measurements were collected at suitable time points in order to generate sensitive, valid and reliable data. |
| 1. NMs physiochemical properties characterization were adequately performed at least ‘as received’, ‘as administered’ and ‘after administration or in situ’. |
| 1. Research personnel were blinded to the study group during data collection and analysis or it was unlikely that lack of blinding significantly affected the results. |
| 1. Cytotoxicity was measured and the test compound did not cause cytotoxicity that significantly affected the results. |
| 1. The statistical methods were clearly described and do not seem inappropriate, unusual or unfamiliar. |
| 1. Are there any other aspects of study design, performance or reporting that influence reliability? |

Identified refinement needs of SciRAPnano v1.0

1. **Reporting quality:**

#12 Solubility and #13 dissolution rate have similar meaning and need to be integrated into one criterion.

1. **Methodological quality:**

#2: *The transformation of the test item or temporal changes of its physicochemical properties has been considered and/or monitored.*

In some cases, the transformation of the NM was considered in the study but exerted strong impact to the study result. For instance, in study 3, strong agglomeration in the dispersion was reported, but it was suspected that the delivered dose was not linear with the nominal dose, indicating the misinterpretation of the result. Hence, the issue “is the NM transformation reported?” and the issue “does the NM transformation affect the result?”, should be evaluated separately.

#3: *The stable dispersion of the test item was created and maintained by appropriate sample preparation method.*

In some cases, the dispersion stability could not be maintained even under optimal test condition owing to the intrinsic (e.g., surface charge) and extrinsic (e.g., interaction with the aqueous media) characteristic of the tested NM. Hence, low dispersion stability does not indicate the poor methodological quality. Instead, the dispersion stability should be fully reported to interpret the study result.

#5: *An appropriate solvent (vehicle) was used that is not expected to interfere with the results of the study at the concentration used. Dispersant/stabilizers was used properly if needed.*

#6: *A solvent (vehicle) control was included. Dispersants/stabilizers were added if needed.*

Even though dispersant/stabilizers may be added to the NM dispersion, the solvent (vehicle) in the dispersion and added dispersant/stabilizers are separated issues. Hence, the appropriateness of solvent and dispersant should be evaluated separately in SciRAPnano criteria. In some cases where the solvent was used appropriately but the added dispersant was not proper, it was difficult to conclude the judgement of two issues (fulfilled or not fulfilled) in one single criterion.

#10: *The dose metrics used were suitable for the test item.*

Dose metrics should be moved to RQ since different metrics used for the exposure level does not impact the study result. Well-reported dose metrics will improve the interpretation of the result.

#11: *The concentrations used were suitable for the test system and investigated endpoints. Transformation or agglomeration of the test item should be considered.*

The gist of this criterion is that, only considering the test system and the endpoint, the concentration is suitable in this specific context (e.g., the concentration is not extremely high leading to evident cytotoxicity which strongly impacts the test system). Even though the (i) reduced (due e.g.to agglomeration or precipitation) delivery of NMs into test systems should be considered or, (ii) dosing and exposure methodologies may need to be adapted when NMs may change form during and after release to the test system (OECD 2012), these above transformation considerations have already been covered by MQ#2.

#12 *The test conditions during and after exposure to the test compound were suitable (media and serum used, cell density, incubation temperature, humidity, CO2 concentration, NMs incompatibilities and interference).*

NMs incompatibilities and interference were newly added aspects compared to the original criterion since the test condition may alter the physicochemical properties of NMs. For instance, the surface area of NM would be affected by the high ionic strength of test-specific culture media, resulting in high particle agglomeration and thus impact the cellular uptake and dosimetry (OECD 2012). However, this aspect has already been covered by the criterion that is related to temporal physicochemical properties changes of NMs (MQ #2).

However, unlike the interference with the test method, NMs interactions with the test condition do not need to be addressed or modified for NMs. Instead, the physicochemical change of NMs caused by NM-condition interaction (e.g., the protein corona formation in the serum medium may strongly influence the uptake and toxicity of the tested NMs(Yin et al. 2015)) need to be analyzed and understood to better interpret the result. Along with the potential amendment of MQ#2, the transformation or other temporal physicochemical changes of the NM should be described. This aspect should be added to RQ criteria.

#13 *Reliable and sensitive tests and/or analytical methods were used for investigating the endpoints, the physicochemical properties of the tested NM or the potential interference has been taken into account.*

The aspect that physicochemical properties of the tested NM may affect and interfere with the test method were newly added aspects compared to the original criterion.

Admittedly, NMs might interfere with assay process and components. In this case, measurements should be taken to avoid or address the test item interference with the test method and thus potential interference does not impact the result. However, this aspect should be separated out from this criterion since the gist of the original criterion is that the test method is suitable for the specific endpoint *per se*. For example, micronucleus assay could be chosen for genotoxicity testing of nano-TiO_2_. However, the potential interference (e.g., NMs interact with the cyto B) is not supposed to be included in this criterion regarding the appropriateness of the chosen method, instead, it should be evaluated in another separated criterion which exclusively requires that the potential interference does not affect the study result.

#16: *NMs physicochemical properties characterization were adequately performed at least ‘as received’, ‘as administered’ and ‘after administration or in situ’.*

There is no clear guidance regarding the physicochemical characterization of NMs in the test system (i.e., in situ) since most of the reporting formats of physicochemical properties are designed for pristine NMs. Hence, unclear guidance without the possibility to be adequately clarified should be excluded. Besides, the reason why this criterion was proposed is that the behavior of the tested NM before, during and after being introduced to the test system should be monitored for understanding the NM behavior in the test system and interpreting the result clearly. Nevertheless, this aspect was partly covered by the criteria regarding transformation (MQ#2) and NM interference (MQ#12,13), which are the two main causes of NM’s changed behavior.

Due to the difficulty in characterizing NM’s properties in the test system, the “black box” regarding the NM’s exact physicochemical behavior/properties within the cell are still poorly understood (ISO 2012). Full NM’s physicochemical characterization “*in situ*” may not be critical, since full characterization of NMs’ physiochemical properties in pristine form and the description of agglomeration/transformation in dispersion, paired with the appropriate experimental design and performance, are probably adequate to conclude the test result could be regarded as “reliable”, even without the thorough understanding the NM behavior in the “black box”.

1. **Identified need for additional NM-specific aspects:**

Cellular uptake or distribution of NMs within the test system should be measured, quantitatively or qualitatively, or inferred from the exposure concentration and particle deposition models and sufficient physicochemical characterization of the NM, if applicable (ISO 2012). This aspect should be added to RQ criteria.

**References**

Beronius A, Molander L, Zilliacus J, Rudén C, Hanberg A. 2018. Testing and Refining the Science in Risk Assessment and Policy (Scirap) Web-Based Platform for Evaluating the Reliability and Relevance of in Vivo Toxicity Studies. 38:1460-1470. <https://doi.org/10.1002/jat.3648>

Comandella D, Gottardo S, Rio-Echevarria IM, Rauscher H. 2020. Quality of Physicochemical Data on Nanomaterials: An Assessment of Data Completeness and Variability. Nanoscale 12:4695-4708. 10.1039/C9NR08323E

Drasler B, Sayre P, Steinhäuser KG, Petri-Fink A, Rothen-Rutishauser B. 2017. In Vitro Approaches to Assess the Hazard of Nanomaterials. NanoImpact 8:99-116. 10.1016/j.impact.2017.08.002

ECHA. Guidance on Information Requirements and Chemical Safety Assessment.

ECHA. 2017. Appendix R7-1 Recommendations for Nanomaterials Applicable to Chapter R7a Endpoint Specific Guidance - Guidance on Information Requirements and Chemical Safety Assessment.ECHA.

EFSA. 2021. Nanomaterials in Reach and Clp - Environment - European Commission.

EFSA, Younes M, Aquilina G, Castle L, Engel K-H, Fowler P, et al. 2021. Safety Assessment of Titanium Dioxide (E171) as a Food Additive. 1831-4732.European Food Ssafety Agency - Panel on Food Additives, Flavourings. <https://doi.org/10.2903/j.efsa.2021.6585>

Elberskirch L, Binder K, Riefler N, Sofranko A, Liebing J, Minella CB, et al. 2022. Digital Research Data: From Analysis of Existing Standards to a Scientific Foundation for a Modular Metadata Schema in Nanosafety. Part Fibre Toxicol 19:1. 10.1186/s12989-021-00442-x

Fernández-Cruz ML, Hernández-Moreno D, Catalán J, Cross RK, Stockmann-Juvala H, Cabellos J, et al. 2018. Quality Evaluation of Human and Environmental Toxicity Studies Performed with Nanomaterials – the Guidenano Approach. Environmental Science: Nano 5:381-397. 10.1039/C7EN00716G

French RA, Jacobson AR, Kim B, Isley SL, Penn RL, Baveye PC. 2009. Influence of Ionic Strength, Ph, and Cation Valence on Aggregation Kinetics of Titanium Dioxide Nanoparticles. Environmental Science & Technology 43:1354-1359. 10.1021/es802628n

Hardy A, Benford D, Halldorsson T, Jeger MJ, Knutsen HK, More S, et al. 2017. Guidance on the Use of the Weight of Evidence Approach in Scientific Assessments. EFSA Journal 15:e04971. 10.2903/j.efsa.2017.4971

ISO. 2012. Iso/Tr 13014:2012(En). In: Nanotechnologies — Guidance on physico-chemical characterization of engineered nanoscale materials for toxicologic assessment. Internet.

Kroll A, Pillukat MH, Hahn D, Schnekenburger J. 2012. Interference of Engineered Nanoparticles with in Vitro Toxicity Assays. Arch Toxicol 86:1123-1136. 10.1007/s00204-012-0837-z

Molander L, Ågerstrand M, Beronius A, Hanberg A, Rudén C. 2015. Science in Risk Assessment and Policy (Scirap): An Online Resource for Evaluating and Reporting in Vivo (Eco)Toxicity Studies. Human and Ecological Risk Assessment: An International Journal 21:753-762. 10.1080/10807039.2014.928104

Nymark P, Grafström R, Noorlander C, Catalán J, Rodriguez-Llopis I, Suárez-Merino B, et al. 2017. Calibrate D5.3 - Document on Quality Criteria for Data. 10.5281/zenodo.3859951

OECD. 2005. Guidance Document on the Validation and International Acceptance of New or Updated Test Methods for Hazard Assessment. Oecd series on testing and assessment Number 34.

OECD. 2012. Guidance on Sample Preparation and Dosimetry for the Safety Testing of Manufactured Nanomaterials. (Series on the Safety of Manufactured Nanomaterials ).OECD.

OECD. 2014. Genotoxicity of Manufactured Nanomaterials : Report of the Oecd Expert Meeting. (Series on the Safety of Manufactured Nanomaterials).

OECD. 2016. Physical-Chemical Properties of Nanomaterials: Evaluation of Methods Applied in the Oecd-Wpmn Testing Programme. (Series on the Safety of Manufactured Nanomaterials).OECD.

OECD. 2017. Test No. 318: Dispersion Stability of Nanomaterials in Simulated Environmental Media. Paris:Organisation for Economic Co-operation and Development.

OECD. 2018. Evaluation of in Vitro Methods for Human Hazard Assessment Applied in the Oecd Testing Programme for the Safety of Manufactured Nanomaterials. (Series on the Safety of Manufactured Nanomaterials).OECD.

OECD. 2019a. Physical-Chemical Decision Framework to Inform Decisions for Risk Assessment of Manufactured Nanomaterials. Series on the Safety of Manufactured Nanomaterials No. 90.

OECD. 2019b. Guiding Principles for Measurements and Reporting for Nanomaterials: Physical Chemical Parameters. (Series on the Safety of Manufactured Nanomaterials).OECD.

OECD. 2019c. Physical-Chemical Parameters: Measurements and Methods Relevant for the Regulation of Nanomaterials. (Series on the Safety of Manufactured Nanomaterials).OECD.

OECD. 2020. Guidance Document for the Testing of Dissolution and Dispersion Stability of Nanomaterials and the Use of the Data for Further Environmental Testing and Assessment Strategies. (Series on Testing and Assessment).OECD.

OECD. 2022. Important Issues on Risk Assessment of Manufactured Nanomaterials. Series on the Safety of Manufactured Nanomaterials No. 103.

Petersen EJ, Henry TB, Zhao J, MacCuspie RI, Kirschling TL, Dobrovolskaia MA, et al. 2014. Identification and Avoidance of Potential Artifacts and Misinterpretations in Nanomaterial Ecotoxicity Measurements. Environmental Science & Technology 48:4226-4246. 10.1021/es4052999

Robinson RLM, Lynch I, Peijnenburg W, Rumble J, Klaessig F, Marquardt C, et al. 2016. How Should the Completeness and Quality of Curated Nanomaterial Data Be Evaluated? Nanoscale 8:9919-9943. 10.1039/C5NR08944A

Roth N, Zilliacus J, Beronius A. 2021. Development of the Scirap Approach for Evaluating the Reliability and Relevance of in Vitro Toxicity Data. 3. 10.3389/ftox.2021.746430

SCENIHR. 2006. The Appropriateness of Existing Methodologies to Assess the Potential Risks Associated with Engineered and Adventitious Products of Nanotechnologies

Stone V, Gottardo S, Bleeker EAJ, Braakhuis H, Dekkers S, Fernandes T, et al. 2020. A Framework for Grouping and Read-across of Nanomaterials- Supporting Innovation and Risk Assessment. Nano Today 35:100941. 10.1016/j.nantod.2020.100941

Taurozzi JS, Hackley VA, Wiesner MR. 2012. Reporting Guidelines for the Preparation of Aqueous Nanoparticle Dispersions from Dry Materials - Version 2.1. NIST SP 1200-1.National Institute of Standards and Technology.

Yin H, Chen R, Casey PS, Ke PC, Davis TP, Chen C. 2015. Reducing the Cytotoxicity of Zno Nanoparticles by a Pre-Formed Protein Corona in a Supplemented Cell Culture Medium. RSC Adv 5:73963-73973. 10.1039/C5RA14870G
