# Supplementary material for: SciRAPnano: a pragmatic and harmonized approach for quality evaluation of in vitro toxicity data to support risk assessment of nanomaterials
Source: Front Toxicol. 2023 Nov 17;5:1319985. doi: 10.3389/ftox.2023.1319985 (PMC10691260; doi:10.3389/ftox.2023.1319985)
Supplement: Supplementary file 2 [file Table2.docx]

**Supporting information - Appendix B**

**Table B1**. Overview of the selected studies in TiO2 case study

| **Study** | **Test item** | **Test system** | **Exposure duration** | **Test method** | **Exposure level** | **Endpoints** |
| --- | --- | --- | --- | --- | --- | --- |
| 1. Multiple endpoints to evaluate pristine and remediated titanium dioxide nanoparticles genotoxicity in lung epithelial A549 cells (Stoccoro et al. 2017) | Pristine TiO2, citrate coated TiO2 and silica coated TiO2 nanoparticles | Human A549 cells | 48h  48h  72h and 48h | CBMN-cyt assay and FISH  Comet assay  Global DNA methylation analysis | 10, 20 and 40 μg/cm2 (32, 64 and 128 μg/ml)  10, 20 and 40 μg/cm2 (32, 64 and 128 μg/ml)  40 μg/cm2 (128 μg/ml) | Genotoxicity |
| 1. Impact of anatase and rutile titanium dioxide nanoparticles on uptake carriers and efflux pumps in Caco-2 gut epithelial cells (Dorier et al. 2015) | Anatase(12nm) and rutile(20nm) TiO2 nanoparticles | Human Caco-2 cells | 6h  6h  6h and 48h | Comet assay  H2-DCF-DA assay  RT-PCR | 50 μg/ml | Genotoxicity,  ROS generation  Impact of TiO2-NPs on nutrient uptake transporters and xenobiotic efflux pumps |
| 1. Role of the crystalline form of titanium dioxide nanoparticles: Rutile, and not anatase, induces toxic effects in Balb/3T3 mouse fibroblasts (Uboldi et al. 2016) | Anatase and rutile TiO2 nanoparticles | Balb/3T3 mouse fibroblasts | 24h  72h | CBMN assay  Cell transformation assay | 10 μg/ml | Genotoxicity  Morphological neoplastic transformation |
| 1. Comparative toxicity assessment of nano- and bulk-phase titanium dioxide particles on the human mammary gland in vitro (Kumar et al. 2020) | Nano-TiO2  Bulk-TiO2 | Human mammary epithelial cells (MCF-7 cells) | 3, 6, 12, 24h  12h  12h | DCFH-DA assay  Comet assay  Annexin V-FITC/PI staining | 1,2,5,10,20,50 and 100μg/ml | ROS generation  Genotoxicity  Apoptosis |
| 1. Effects of Titanium Dioxide Nanoparticles on the Hprt Gene Mutations in V79 Hamster Cells (Kazimirova et al. 2020) | TiO2 nanoparticles | Chinese hamster lung (V79) fibroblasts | 24h | Hprt Mammalian Gene Mutation Assay | 3,15 and 75μg/cm2 | Genotoxicity |
| 1. Effects of differently shaped TiO2NPs (nanospheres, nanorods and nanowires) on the in vitro model (Caco-2/HT29) of the intestinal barrier (García-Rodríguez et al. 2018) | TiO2 (nanospheres, nanorods and nanowires) | Human Caco-2/HT29 cells | 24 and 48h | Trans-epithelial electrical resistance (TEER)  RT-PCR  Comet assay | 12.5, 50, 150 and 350μg/ml  50 and 150μg/ml  12.5, 50, 150 and 350μg/ml | Intestinal epithelial barrier’s integrity  Gene expression of tight junction components  Genotoxicity |
| 1. Effect of titanium dioxide nanoparticles on DNA methylation in multiple human cell lines (Pogribna et al. 2020) | TiO2 nanoparticles | Human Caco-2, HepG2, NL20, and A-431 cells | 24h and 72h | ELISA-based colorimetric assay  Methyl-Profiler DNA Methylation PCR System | 100μg/ml | DNA methylation (global and gene-specific) |
| 1. Assessment of the Influence of Crystalline Form on Cyto-Genotoxic and Inflammatory Effects Induced by TiO2 Nanoparticles on Human Bronchial and Alveolar Cells (Fresegna et al. 2021) | Anatase and rutile TiO2 nanoparticles | Human A549 and BEAS-2B cells | 30 min, 2 h and 24 h  2 and 24 h | LDH assay  Fpg modified comet assay | 1, 5, 10, 20, and 40 μg/ml | Membrane damage  Genotoxicity |
| 1. Titanium Dioxide Nanoparticles Alter the Cellular Phosphoproteome in A549 Cells (Biola-Clier et al. 2020) | TiO2 nanoparticles | Human A549 cells | 24h | Tandem Mass Spectrometry  MS/MS Spectra Interpretation, Statistics, and Data Mining  Electron Microscopy | 100μg/ml, corresponds to 17μg TiO2/cm2, i.e., 0.11 ng TiO2/cell | Cellular Phosphoproteome |
| 1. Comparative study of the transcriptomes of Caco-2 cells cultured under dynamic vs. static conditions following exposure to titanium dioxide and zinc oxide nanomaterials (Kulthong et al. 2021) | Nano-TiO2 (and Nano-ZnO) | Human Caco-2 cells | 6h | Affymetrix microarray | Gut-on-chip: 100μg/mL  Transwell: 10 and 50μg/mL: | Transcriptomes |
| 1. Vasomotor dysfunction in human subcutaneous arteries exposed ex vivo to food-grade titanium dioxide (Jensen et al. 2018) | food-grade TiO2 nanoparticles | Human subcutaneous arteries | 30 min and 18 h | Myograph  RT-PCR | 14 or 140 μg/ml | Vasomotor responses  Gene expression of 5-HT receptors, endothelin receptors and markers of endothelial activation |

**References**

Biola-Clier M, Gaillard J-C, Rabilloud T, Armengaud J, Carriere M. 2020. Titanium Dioxide Nanoparticles Alter the Cellular Phosphoproteome in A549 Cells. Nanomaterials (Basel) 10:E185. 10.3390/nano10020185

Dorier M, Brun E, Veronesi G, Barreau F, Pernet-Gallay K, Desvergne C, et al. 2015. Impact of Anatase and Rutile Titanium Dioxide Nanoparticles on Uptake Carriers and Efflux Pumps in Caco-2 Gut Epithelial Cells. Nanoscale 7:7352-7360. 10.1039/c5nr00505a

Fresegna AM, Ursini CL, Ciervo A, Maiello R, Casciardi S, Iavicoli S, et al. 2021. Assessment of the Influence of Crystalline Form on Cyto-Genotoxic and Inflammatory Effects Induced by Tio2 Nanoparticles on Human Bronchial and Alveolar Cells. Nanomaterials 11:253. 10.3390/nano11010253

García-Rodríguez A, Vila L, Cortés C, Hernández A, Marcos R. 2018. Effects of Differently Shaped Tio2nps (Nanospheres, Nanorods and Nanowires) on the in Vitro Model (Caco-2/Ht29) of the Intestinal Barrier. Part Fibre Toxicol 15:33. 10.1186/s12989-018-0269-x

Jensen DM, Skovsted GF, Lykkesfeldt J, Dreier R, Berg JO, Jeppesen JL, et al. 2018. Vasomotor Dysfunction in Human Subcutaneous Arteries Exposed Ex Vivo to Food-Grade Titanium Dioxide. Food and Chemical Toxicology 120:321-327. 10.1016/j.fct.2018.07.015

Kazimirova A, El Yamani N, Rubio L, García-Rodríguez A, Barancokova M, Marcos R, et al. 2020. Effects of Titanium Dioxide Nanoparticles on the Hprt Gene Mutations in V79 Hamster Cells. Nanomaterials 10:465. 10.3390/nano10030465

Kulthong K, Hooiveld GJEJ, Duivenvoorde LPM, Miro Estruch I, Bouwmeester H, van der Zande M. 2021. Comparative Study of the Transcriptomes of Caco-2 Cells Cultured under Dynamic Vs. Static Conditions Following Exposure to Titanium Dioxide and Zinc Oxide Nanomaterials. Nanotoxicology 15:1233-1252. 10.1080/17435390.2021.2012609

Kumar S, Hussain A, Bhushan B, Kaul G. 2020. Comparative Toxicity Assessment of Nano- and Bulk-Phase Titanium Dioxide Particles on the Human Mammary Gland in Vitro. Human & Experimental Toxicology 39:1475-1486. 10.1177/0960327120927448

Pogribna M, Koonce NA, Mathew A, Word B, Patri AK, Lyn-Cook B, et al. 2020. Effect of Titanium Dioxide Nanoparticles on DNA Methylation in Multiple Human Cell Lines. Nanotoxicology 14:534-553. 10.1080/17435390.2020.1723730

Stoccoro A, Di Bucchianico S, Coppedè F, Ponti J, Uboldi C, Blosi M, et al. 2017. Multiple Endpoints to Evaluate Pristine and Remediated Titanium Dioxide Nanoparticles Genotoxicity in Lung Epithelial A549 Cells. Toxicology Letters 276:48-61. 10.1016/j.toxlet.2017.05.016

Uboldi C, Urbán P, Gilliland D, Bajak E, Valsami-Jones E, Ponti J, et al. 2016. Role of the Crystalline Form of Titanium Dioxide Nanoparticles: Rutile, and Not Anatase, Induces Toxic Effects in Balb/3t3 Mouse Fibroblasts. Toxicology in Vitro 31:137-145. 10.1016/j.tiv.2015.11.005
