# Supplementary material for: SciRAPnano: a pragmatic and harmonized approach for quality evaluation of in vitro toxicity data to support risk assessment of nanomaterials
Source: Front Toxicol. 2023 Nov 17;5:1319985. doi: 10.3389/ftox.2023.1319985 (PMC10691260; doi:10.3389/ftox.2023.1319985)
Supplement: Supplementary file 4 [file Table3.docx]

**Supporting information – Appendix C**

**Table C1**. SciRAPnano criteria and guidance for assessing methodological quality of *in vitro* toxicity studies of nanomaterials.

| **Criteria** | **Guidance** |
| --- | --- |
| **Test item and controls** |  |
| 1. The test item or mixture was unlikely to contain any impurities that may significantly have affected the results of the study. | Purity of the test item, or the composition of substances in a mixture can potentially affect study results. Purity and composition is also an important aspect to consider in terms of the relevance of the test item to the compound being risk assessed. Ideally, in the case of single compounds, the test chemical should be of the highest available purity.  Here you can also consider other aspects of the test item, such as physicochemical properties or the composition and characteristics of a nanomaterial, which may affect study results.  How to judge this criterion:  Fulfilled – The test item has been clearly identified and characterized and is of sufficient purity. In cases of mixtures, the composition of substances is well characterized and their individual purities are sufficient. There is no suspicion that impurities or any other physicochemical properties of the test item has affected the results of the study.  Partially fulfilled – The purity of the test item or mixture has not been described but it is unlikely that impurities or other physicochemical properties of the test item are present that would significantly affect the results of the study.  Not fulfilled – The test item or mixture is likely to contain impurities or have physicochemical properties that can affect study results. |
| 1. A dispersion of the test item was created by appropriate sample preparation method. | Sample preparation is widely recognized as one of the most critical steps towards successful characterization and subsequent testing of nanomaterials, as it is expected to affect agglomeration or aggregation, which in turn affects the following physiochemical properties characterization, cell exposure, cytotoxicity, dose selection and genotoxicity (ECHA 2017). Preparation protocols may be strongly dependent on the nature of the sample to be dispersed. Thus, selected sample preparation procedure should be justified and sufficiently reported. The homogenous dispersion and consistent concentration should be created by appropriate sample preparation procedure.  Common issues to be considered regarding sample preparation include storage, colloidal and chemical stability of the tested nanomaterial, the chemical composition of the test media (which affects particle aggregation/agglomeration), characterization of stock dispersions and characterization of samples (prepared from stock dispersions) prior to administration/testing. The Guidance on Sample Preparation and Dosimetry for the Safety Testing of Manufactured Nanomaterials OECD No. 36 13 ENV/JM/MONO(2012)40 has the best practice (OECD 2012).  Instead of “solution”, the term “dispersion” is recommended to be used in nanomaterials due to the fact that nanomaterials colloids are *dispersed* rather than *dissolved* in the medium (OECD 2012).  How to judge this criterion:  Fulfilled – A dispersion was created following appropriate sample preparation method.  Partially fulfilled – The dispersion of the test item at the concentration used has not been described but based on the methods description it is likely that the test item was stably dispersed at the concentrations used.  Not fulfilled – It is suspected that the test item was not stably dispersed in the test system or it is assumed that the test item was unstably dispersed at the concentration used. |
| 1. The transformation of the test item or temporal changes of its physiochemical properties is not expected to affect the result. | Transformations of NMs due to the time effect (aging), storage of the dispersions under different conditions and capping agent used to stabilize them, has been suggested as one of the most significant contributors to the contradictory in vitro toxicity results observed in the literature for identical NMs.  In some cases, the temporal changes in NMs' properties in different media are inevitable, even under optimal conditions. It may modulate NMs’ toxicity, by altering their dissolution rate or agglomeration state which affects bioavailability, leading to discrepancies of administered vs. delivered dose. Hence, aging needs to be assessed in parallel with the assessment of effects. For instance, the NMs' physicochemical parameters such as size/agglomeration, degradation, surface charge and dissolution throughout the duration of the study are periodically monitored to ensure that any changes can be accounted for in the data interpretation and analysis (Izak-Nau et al. 2015).  Moreover, the test condition may alter the physiochemical properties of NMs. For instance, the surface area of NM would be affected by the high ionic strength of test-specific culture media, resulting in high particle agglomeration (French et al. 2009).  Fulfilled: There is no suspicion that transformation or temporal changes of physicochemical properties of the test item have significantly affected the study result.  Partly fulfilled: It is suspected that the transformation or temporal changes of the physio-chem properties of test item but it is unlikely that transformation of the test NM is present that significantly impact the results of the study.  Not fulfilled: The transformation of the test NM is likely to happen and may affect study results. |
| 1. An appropriate solvent (vehicle) was used that is not expected to interfere with the results of the study at the concentration used. | A solvent or vehicle is “any agent which serves as a carrier used to mix, disperse, or solubilize the test item or reference item to facilitate the administration/application to the test system” (OECD 1998). The choice of solvent will be determined by the solubility of the test item, as well as the test system used.  The test item is usually dissolved in ethanol or DMSO as vehicle. The final solvent concentration in the test system is commonly <=1% (OECD 2018).  How to judge this criterion:  Fulfilled – ethanol, DMSO or water or another common and historically well-characterized solvent. The concentration of the solvent was appropriate and it is not expected to interfere with the results.  Partially fulfilled – the solvent was not well characterized or is not commonly used in this context, or the solvent/dispersant concentration was not clearly stated, but there are no obvious concerns that it interferes with the results.  Not fulfilled – the solvent used was clearly interfering with the results or the solvent concentration was too high. |
| 1. A solvent (vehicle) control was included. | A solvent (vehicle) control that is not treated with the test item should always be included as it is critical for determining treatment-related effects.  Control samples should be handled in the same way as treated samples.  How to judge this criterion:  Fulfilled – An untreated solvent control was included.  Partially fulfilled – It is not explicitly stated that an untreated solvent control was included, but it is likely that it was included.  Not fulfilled – no untreated solvent control was included. |
| 1. The dispersant/stabilizer used is not expected to interfere with the results of the study. | The usage of dispersant/stabilizer should be justified practical reasons. The type and amount of dispersant should be proper, not changing the shape and agglomeration state of NMs in the medium or exaggerating the toxicity (additive or synergistic effect) of the test substance (Andreani et al. 2020; Hartmann et al. 2015). Dispersant similar to what would be found in the target tissue, such as bronchoalveolar lavage fluid (BALF) or mimic BALF for inhalation studies, should be considered first.  Besides, if a dispersant or stabilizer was used during sample preparation, it should be added to the solvent control as the dispersant alone (e.g., Pluronic dispersants) can induce adverse effects, particularly upon sonication.  Fulfilled: The type and amount of dispersant/stabilizer used is justified appropriate and expected not to interfere with the study result. Or the dispersant/stabilizer was not used.  Partly fulfilled: the type of the dispersant/stabilizer is not commonly used in this context, or the type and amount is not clearly stated. But there are no obvious concerns that it interferes with the results.  Not fulfilled: the dispersant/stabilizer used was clearly interfering with the results or the concentration was too high. |
| 1. An appropriate positive control was included, and the expected result was observed from this treatment. | A positive control is a substance known to induce a positive response in a specific test system. Alternatively, it is defined as a sample or replicate “containing all components of a test system and treated with a substance known to induce a positive response” (e.g. OECD TG No. 437, 439, 491, 494). The positive control is included and monitored to ensure that the test system is performing as expected and able to detect effects in the intended endpoints. Thus, the positive control must be carefully chosen so that the sensitivity is appropriately captured relative to the toxicity of the test item.  Besides chemical controls, NM-based controls are recommended to confirm the sensitivity of the techniques for NMs, to benchmark the NM biological effects, and to evaluate potential NM interferences with the assay. The appropriateness of the positive control will also depend on the system and test model used, including different models available for the same type of test.  Positive control samples should be handled in the same way as treated samples.  How to judge this criterion:  Fulfilled – An appropriate positive control was included. The expected effect was observed in the positive control and the positive control treatment was appropriate in terms of similar mechanism as the test item and effects observed for the measured endpoints.  Partially fulfilled – It is likely but not explicitly stated that a positive control was included, or the appropriateness of the positive control is not fully established but it is likely appropriate, or a historical positive control was referred to.  Not fulfilled – no positive control was included, or the expected effect was not observed from the positive control treatment. |
| **Test system** |  |
| 1. A reliable and sensitive test system (cell line / cells / tissue / organ /embryo / sub-cellular fractions) with metabolic competence, if relevant, was used for investigating the test item and endpoints. | The choice of test system (cell line / cells / tissue / organ /embryo / sub-cellular fractions) is based on a number of considerations, including knowledge regarding metabolism and mode of action.  Reliability, in this context, refers to whether the test system has been shown to generate reproducible results for the type of endpoints investigated.  The sensitivity of the test system relates to the ability to detect changes in the endpoints investigated in the model.  How to judge this criterion:  Fulfilled – The test system used is well-established or the reliability and sensitivity of the test system is clearly described. In case metabolism of the test item is relevant for the toxicity, the metabolic competence of the test system is well-established or it is clearly stated that the test system has the appropriate metabolic competence.  Partially fulfilled – It is likely that the test system is reliable and sensitive but it is not a well-established system and reliability and sensitivity of the test system is not clearly described. In case metabolism of the test item is relevant for the toxicity, it is likely that the test system has the appropriate metabolic competence, although it is not clearly stated.  Not fulfilled – there is available information that indicates that the test system is either insensitive or clearly unreliable for studying the test item or for investigating the endpoints considered. Or the expected outcome is lacking from positive control, if included. Or the test system lacks metabolic competence required to assess the toxicity of the test item. |
| 1. Conditions for cultivation and/or maintenance of the cell line / cells / tissue / organ /embryo (incubation temperature, humidity, CO2 concentration, media used, number of cell passages, control of contamination) were appropriate. | Conditions for cultivation and/or maintenance of the cell line / cells / tissue / organ /embryo should be appropriate. This includes e.g. incubation temperature, humidity, CO2 concentration, media used, number of cell passages, control of contamination. Guidance for Good Cell Culture Practice (GCCP) describes the best practice (Coecke et al. 2005).  How to judge this criterion:  Fulfilled – Conditions for cultivation and/or maintenance have been fully described and were in line with standard recommendations for the test system.  Partially fulfilled – Most of the conditions for cultivation and/or maintenance were described and are in line with standard recommendations for the test system. Others deviated from standard recommendations or were not reported.  Not fulfilled – Most of the conditions for cultivation and/or maintenance were not described or were not in line with standard recommendations for the test system. |
| **Administration of the test item** |  |
| 1. The duration of exposure was suitable for the test system and investigated endpoints. | The duration of exposure should be adapted to the test system and endpoint.  How to judge this criterion:  Fulfilled – the duration of exposure of the test item is clearly suitable for the test system and endpoint.  Partially fulfilled – the duration of exposure of the test item is suitable for the test system and endpoint with some deviation.  Not fulfilled - the duration of exposure of the test item is not suitable for the test system and endpoint. |
| 1. The concentrations used were suitable for the test system and investigated endpoints. | The concentrations used should be adapted to the test system and endpoint.  Since NMs may change form during and after release to the test system, dosing and exposure methodologies may need to be adapted if material modifications are reasonably anticipated (OECD 2012).  How to judge this criterion:  Fulfilled – the concentrations of the test item are clearly suitable for the test system and endpoint.  Partially fulfilled – the concentrations of the test item are suitable for the test system and endpoint with some deviation.  Not fulfilled - the concentrations of the test item are not suitable for the test system and endpoint. |
| 1. The test conditions during and after exposure to the test item were suitable (media and serum used, cell density, incubation temperature, humidity, CO2 concentration) | Test conditions during and after exposure to the test item should be appropriate. This includes e.g. media and serum used, cell density, incubation temperature, humidity, CO2 concentration. Inappropriate oxygen concentration and temperature may also alter the agglomeration chemistry of NMs. These possibilities needs to be taken into account when selecting the test condition (OECD 2012).  How to judge this criterion:  Fulfilled – Test conditions during and after exposure to the test item have been fully described and were appropriate.  Partially fulfilled – Most of the test conditions during and after exposure to the test item were described and were appropriate. Others deviated from standard recommendations.  Not fulfilled – Most of the conditions test conditions during and after exposure to the test item were not described or were not in line with standard recommendations. |
| **Data collection and analysis** |  |
| 1. Reliable and sensitive tests and/or analytical methods were used for investigating the endpoints. | The reliability of the tests and methods refers to whether they are known to generate reproducible results for the type of endpoints investigated, e.g. if the methods have been validated across different laboratories. The sensitivity of the methods relates to the ability to detect changes in the endpoints investigated.  The applicability of the method used in NMs toxicity testing should be taken into account. For example, the bacterial reverse mutation test may not be appropriate for the investigation of the genotoxicity of nanomaterials*.* Find the most relevant OECD TG in *Guidelines considered most relevant for investigating health effects of nanomaterials* (OECD 2009)  How to judge this criterion:  Fulfilled – there is no information that suggests that the test methods are insensitive or unreliable in this context.  Partially fulfilled – it is suspected that one or more of the methods applied may be insensitive or unreliable.  Not fulfilled – there is available information that indicates that one or more of the methods applied is either insensitive or clearly unreliable for studies of the test item or for investigating the endpoints considered. Or the expected outcome is lacking from positive controls, if included, indicating that the methods is insensitive. |
| 1. The test item is not expected to interfere with the test method so as to affect the study result. | The physiochemical properties (e.g., high adsorption capacity, optical properties, surface charge, and catalytic activities) of the tested NM might interfere the chosen test method. For example, Double-Strand Breaks (DSB) assay could be used in SWCNTs genotoxicity testing. However, if NMs with autofluorescence were tested, they would interfere with the quantification of foci.  The test methods need to be adapted for NMs specifically in order to avoid interference. For example, OECD Test Guidelines not appropriate for highly insoluble substances may be applicable for nanomaterials with specific adaptation.  Fulfilled- The potential interference of the test item with the test method was addressed or justified unlikely to affect the study result.  Partially fulfilled- The potential interference of the test item with the test method was not reported but expected unlikely to happen.  Not fulfilled- There is available information that indicates the test method could not be performed accurately due to the potential interference of the test item which affects the result. |
| 1. Sufficient numbers of replicates or repetitions of the experiment were used to generate reliable and valid results. | Sample size should be large enough to ensure sufficient statistical power to detect any effects in the endpoints measured.  How to judge this criterion:  Fulfilled – a sufficient number of replicates or repetitions of the experiment were included  Partially fulfilled – a lower than usual number of replicates were used, which may have caused lower sensitivity/statistical power of the study.  Not fulfilled – the number of replicates was clearly insufficient |
| 1. Measurements were collected at suitable time points in order to generate sensitive, valid and reliable data. | The time points for the measurements should be adapted to the test system and endpoint.  How to judge this criterion:  Fulfilled – the time points for the measurements are clearly suitable for the test system and endpoint.  Partially fulfilled – time points for the measurements are suitable for the test system and endpoint with some deviation.  Not fulfilled - time points for the measurements are not suitable for the test system and endpoint. |
| 1. Cytotoxicity was measured and the test item did not cause cytotoxicity that significantly affected the results. | Cytotoxicity might have significantly affected results and conclusions should only be made based on conditions (concentration of test item and exposure duration) that does not cause significant cytotoxicity.  How to judge this criterion:  Fulfilled – cytotoxicity was measured and the test item did not cause cytotoxicity at the relevant concentrations and exposure time.  Partially fulfilled – cytotoxicity was measured and the test item cause minor cytotoxicity at the relevant concentrations and exposure time that did not affect the results.  Not fulfilled - Cytotoxicity was not measured. Or cytotoxicity was measured and the test item caused cytotoxicity at the relevant concentrations and exposure time. |
| 1. The statistical methods were clearly described and do not seem inappropriate, unusual or unfamiliar. | The choice of statistical analyses will depend on the type of study and the nature of the endpoints measured.  OECD test guidelines and corresponding guidance documents provide some recommendations for statistical tests (e.g. OECD 2002a,b) as well as for considerations to be made in statistical analyses of different types of tests.  In general, normality of the data should have been checked and the choice of parametric or non-parametric tests should have been based upon that result.  How to judge this criterion:  Fulfilled – the statistical methods have been clearly described and do not seem inappropriate, unusual or unfamiliar.  Partially fulfilled – unusual or unfamiliar methods were applied in the statistical analyses but do not seem clearly inappropriate.  Not fulfilled – no statistical tests were used, or the tests used are clearly inappropriate for the study type and/or endpoints measured. |
|  |  |
| 1. Are there any other aspects of study design, performance or reporting that influence reliability? | In this section any additional factors of the study design or conduct that are not covered by the criteria above and that the evaluator considers may increase or decrease reliability of the study should be considered. These may vary on a case-by-case basis and can for example include, but are not limited to, factors such as:  If the test method has been validated, e.g. by assessment of repeatability within a laboratory and reproducibility of the method at multiple laboratory sites.  If there is concern that the potential contamination such as endotoxin presence within the test sample may have influenced the study results or are not sufficiently characterized.  If there is concern that the extra substances (e.g., immune activator, metabolizes inhibitor etc.) added to the test system may impact the effect on particle dispersion and toxicity.  If declarations of conflict of interest or sources of funding raise concern of possible bias or are missing.  Note: this criterion is not included in the color profile read-out for the study generated in the SciRAP tool. Comments made here will be shown in the excel file with the color profile for the study and should be considered in parallel with the color profile when conducting the categorization of study reliability. |

**References**

Andreani T, Fernandes PMV, Nogueira V, Pinto VV, Ferreira MJ, Rasteiro MG, et al. 2020. The Critical Role of the Dispersant Agents in the Preparation and Ecotoxicity of Nanomaterial Suspensions. Environ Sci Pollut Res Int 27:19845-19857. 10.1007/s11356-020-08323-0

Coecke S, Balls M, Bowe G, Davis J, Gstraunthaler G, Hartung T, et al. 2005. Guidance on Good Cell Culture Practice. A Report of the Second Ecvam Task Force on Good Cell Culture Practice. Alternatives to laboratory animals : ATLA 33:261-287. 10.1177/026119290503300313

ECHA. 2017. Appendix R7-1 Recommendations for Nanomaterials Applicable to Chapter R7a Endpoint Specific Guidance - Guidance on Information Requirements and Chemical Safety Assessment.ECHA.

French RA, Jacobson AR, Kim B, Isley SL, Penn RL, Baveye PC. 2009. Influence of Ionic Strength, Ph, and Cation Valence on Aggregation Kinetics of Titanium Dioxide Nanoparticles. Environmental Science & Technology 43:1354-1359. 10.1021/es802628n

Hartmann NB, Jensen KA, Baun A, Rasmussen K, Rauscher H, Tantra R, et al. 2015. Techniques and Protocols for Dispersing Nanoparticle Powders in Aqueous Media—Is There a Rationale for Harmonization? Journal of Toxicology and Environmental Health, Part B 18:299-326. 10.1080/10937404.2015.1074969

Izak-Nau E, Huk A, Reidy B, Uggerud H, Vadset M, Eiden S, et al. 2015. Impact of Storage Conditions and Storage Time on Silver Nanoparticles' Physicochemical Properties and Implications for Their Biological Effects. RSC Adv 5:84172-84185. 10.1039/C5RA10187E

OECD. 2009. Guidance Manual for the Testing of Manufactured Nanomaterials: Oecd’s Sponsorship Programme; First Revision. (Series on the Safety of Manufactured Nanomaterials ).OECD.

OECD. 2012. Guidance on Sample Preparation and Dosimetry for the Safety Testing of Manufactured Nanomaterials. (Series on the Safety of Manufactured Nanomaterials ).OECD.

OECD. 2018. Guidance Document on Good in Vitro Method Practices (Givimp). doi:<https://doi.org/10.1787/9789264304796-en>
